# Multifaceted biomedical applications of biogenic titanium dioxide nanoparticles fabricated by marine actinobacterium *Streptomyces vinaceusdrappus* AMG31

**DOI:** 10.1038/s41598-025-00541-1

**Published:** 2025-06-23

**Authors:** Ahmed Ghareeb, Amr Fouda, Rania M. Kishk, Waleed M. El Kazzaz

**Affiliations:** 1 Botany and Microbiology Department, Faculty of Science, Suez Canal University, Ismailia, 41522 Egypt; 2 Botany and Microbiology Department, Faculty of Science, Al-Azhar University, Nasr City, 11884 Cairo Egypt; 3 Microbiology and Immunology Department, Faculty of Medicine, Suez Canal University, Ismailia, 41522 Egypt

**Keywords:** Green synthesis, TiO_2_-NP, Marine actinobacteria, Biomedical applications, Biotechnology, Chemical biology, Microbiology, Materials science, Nanoscience and technology

## Abstract

The biomass filtrate of marine actinobacterium, *Streptomyces vinaceusdrappus* AMG31, was utilized as a biocompatible and biocatalyst for titanium dioxide nanoparticles (TiO₂-NPs) synthesis. Characterization revealed well-dispersed, spherical structures with high crystallinity in the anatase phase, with sizes from 10 to 50 nm. The biosynthesized TiO₂-NPs demonstrated potent antioxidant activity with maximum DPPH and ABTS radical scavenging percentages of 94.6% and 88.2% at 1000 µg/ml, with IC₅₀ values of 11.1 and 14.36 µg/ml, respectively. TiO₂-NPs exhibited moderate wound healing activity with 66.6% wound closure compared to controls (62.6%) after 48 h. The hemocompatibility assessment revealed minimal hemolytic activity (1.9% at 1000 µg/ml) and modest anticoagulant effects in PT (14.2 s) and PTT (43 s) at 75 µg/ml. Moreover, TiO₂-NPs displayed selective cytotoxicity towards cancer cells (Caco-2 and PANC-1) with IC₅₀ values of 74.1 ± 0.7 and 71.04 ± 1.2 µg/ml, respectively, while showing lower toxicity towards normal WI38 cells (IC₅₀ 153.1 ± 1.01 µg/ml). The nanoparticles demonstrated significant antidiabetic potential through α-amylase and α-glucosidase inhibition (IC₅₀ 69.3 and 40.81 µg/ml, respectively). Notably, TiO₂-NPs exhibited potent antibacterial activity against Gram-positive bacteria, particularly *Enterococcus faecalis* (37 ± 0.1 mm inhibition zone vs. 28 ± 0.1 mm for gentamicin) and Gram-negative bacteria, especially *E. coli* (29 ± 0.1 mm vs. 22 ± 0.2 mm for gentamicin), with low MIC/MBC values (12.5/25 µg/ml for Gram-positive and 6.25/12.5 µg/ml for *E. coli*). The nanoparticles demonstrated superior antifungal activity compared to fluconazole against *Penicillium glabrum* (45 ± 0.1 mm vs. 38 ± 0.1 mm), *Aspergillus niger* (37 ± 0.2 mm vs. 36 ± 0.1 mm), and *Candida albicans* (30 ± 0.3 mm vs. 26 ± 0.3 mm). Furthermore, TiO₂-NPs showed remarkable antibiofilm activity against bacterial (90.8–98.2% inhibition) and fungal (97.3% inhibition for *C. albicans*) biofilms at 75% MBC/MFC concentrations. The actinobacterial TiO₂-NPs’ biological activity profile, in conjunction with their biocompatibility, selective cytotoxicity, and minimal hemolytic activity, positions the actinobacterial TiO₂-NPs as promising candidates for various biomedical applications.

**Supplementary Information:**

The online version contains supplementary material available at 10.1038/s41598-025-00541-1.

## Introduction

The rapid advancements in nanotechnology and nanoscience have generated immense interest and demand for nanoparticles (NPs) in recent years^1^. The key driving factor behind the increasing adoption of NPs is their high surface area-to-volume ratio and elevated surface energy, which make them versatile and suitable for a broad spectrum of applications, including environmental remediation, electronics, scientific research, and the medical field^2^. Therefore, the demand for NPs has been steadily increasing, with scientists and engineers exploring new ways to leverage the exceptional features of these nanomaterials to address^3^. TiO_2_, commonly referred to as titania, is a naturally derived oxide of the element titanium, insoluble in water, and has a remarkably high refractive index of 2.4^4^, making it a valuable white pigment with distinctive light-interacting and electromagnetic characteristics. TiO_2_ can exist in either an amorphous or crystalline state. The crystalline form of TiO_2_ is found in three main polymorphs: anatase (3.2 eV band gap), rutile (2.96 eV band gap), and brookite (3.02 eV band gap) existing in forms ranging from zero-dimensional to three-dimensional structures, all of which hold promising applications in biotechnology^5^. Titanium dioxide nanoparticles (TiO_2_-NPs) are favored over other metal oxides for various applications due to their photocatalytic capabilities, affordability, high abundance, potent oxidizing properties, and superior chemical stability^6^. The smaller size and enhanced reactivity of TiO_2_-NPs further contribute to their attractiveness^7^. Their unique ability to absorb ultraviolet radiation coupled with a high refractive index contributes to their usage, imparting a pure white hue and intense coloration with remarkable opacity^8^.

The synthesis of TiO_2_-NPs can be achieved through various routes, including chemical, physical, and biological approaches^9^. While the chemical methods offer a rapid synthesis process, they are often criticized for their environmental impact due to the extensive use of chemical agents^10,11^. In contrast, physical methods like ball milling and physical vapor deposition^12^, although effective, are notorious for their high energy requirements and bear higher costs^11^. Recognizing these limitations, the biological synthesis route emerges as a promising alternative, harnessing the power of plants and microorganisms to produce TiO_2_-NPs in an eco-friendly manner while ensuring biocompatibility, making them highly suitable for medical applications^13^. The following table (Table 1) compares TiO_2_-NPs synthesized by the biogenic approach and those formed by chemical and physical methods.

**Table 1:** Comparison study between TiO_2_-NPs formed by green approach and those synthesized by chemical and physical methods.

Criterion	Biosynthetic approach	Conventional approaches	References
NPs quality	High purity, uniformly homogenous particle size	Fluctuating quality, potential impurities	^111–114^
Yield	High yield with genetically modified organisms with optimized conditions	Moderate to low yield	
Potential scalability	Efficiently scalable with bioreactors	Limited scalability due to sophisticated processes	
Biotherapeutic application	Adequate efficiency for targeted drug delivery and diagnostic purposes	Restricted functionality, often less specialized	

Microbial-derived TiO_2_-NPs effectively alleviate oxidative stress and the risks of cellular injury by neutralizing various free radicals and reactive oxygen species (ROS) through their unique surface functional groups^14^. The biosynthesized triangular TiO_2_-NPs by *Tricoderma citrinoviride* exhibited superior DPPH free radical scavenging abilities when compared to gallic acid at 50 to 100 µg/ml^15^. Similarly, *Tinospora cordifolia* derived TiO_2_-NPs showed 90% DPPH scavenging activity^16^. Also, *Lawsonia inermis* leaf extract-based synthesized TiO_2_-NPs exhibited oxidative stress, which was measured by hydrogen-induced hemolysis reduction of 82% at concentrations of 5-100 mg/ml^17^.

TiO_2_-NPs possess photocatalytic antimicrobial activity when irradiated with UV light of wavelength less than 385 nm, and their activity is also dependent on the thickness of the cell surfaces of the microbes^18^. For instance, *Staphylococcus aureus* and methicillin-resistant *Staphylococcus aureus* colonies were reduced by 77% and 97%, respectively, using a plate coating technique with spherical TiO_2_-NPs obtained from *S. cerevisiae*^19^. Another, a 23 nm TiO_2_-NP produced by the bacterium *S. spinosa*, exhibited antibacterial properties against *S. aureus* and antifungal activity against *C. albicans*^20^. Also, TiO_2_-NP measured 70 nm and spherical in shape, synthesized from *Streptomyces* sp. HC1 was found to have a moderate antifungal effect against *A.niger* while inhibiting the biofilm development of *Pseudomonas aeruginosa*^21^.

Due to the inorganic character of TiO_2_-NPs, targeted cancer therapies can selectively kill cancerous cells through ROS, which exploits the p53-dependent apoptotic pathway. ROS induces cytochrome c release through the damaging of the mitochondrial membrane, thereby triggering the intrinsic apoptosis pathway leading to optimal cell death^22,23^. In a research study, Doxorubicin was incorporated into TiO_2_-NPs forming DOX-TiO_2_ NPs, which showed cytotoxicity to SMMC–7721 hepatocarcinoma cell line by MTT assay^24^. Another research investigated folic acid conjugated TiO_2_-NPs against MG63 osteosarcoma cells where the conjugated NPs reduced cell volume and caused chromatin condensation and showed two times more cytotoxicity than the unconjugated NPs^24^. Additionally, a mycofabricated TiO_2_-NP from *Aspergillus niger* showed potent anticancer activity against the MCF-7 and HepG-2 cancer cell lines at 97.35% and 97.71%, respectively, while having good viable biocompatibility at the concentration of 1000 µg/ml^25^. It was also demonstrated that bacterial anatase TiO_2_-NP produced by *Exiguobacterium aestuarii* reduced cell viability of HeLa and SiHa cervical cancer cell lines by 80% at 100 µg concentration^26^.

Recognizing the potential of TiO_2_-NPs, this study aims to systematically biosynthesize and comprehensively characterize TiO_2_-NPs using the marine actinobacterium *Streptomyces vinaceusdrappus* AMG31, investigating their multifaceted biomedical capabilities.

## Results and discussion

### Biosynthesis and characterization of TiO_2_-NP

**Fig. 1:**
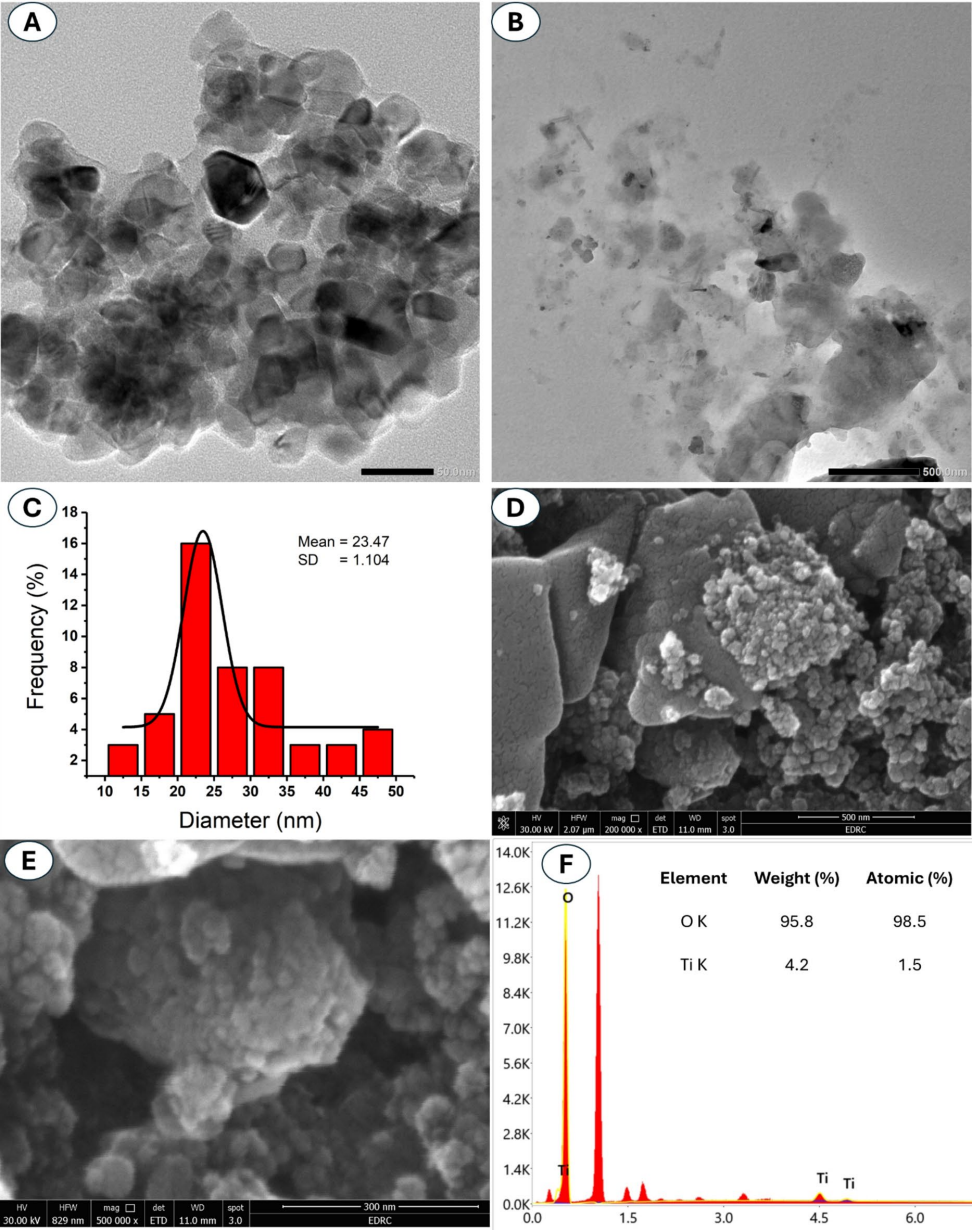
Characterization of actinobacterial-mediated biogenic synthesis of TiO_2_-NPs. (**A**, **B**) TEM images, (**C**) size distribution, (**D**, **E**) the SEM analysis, and (**F**) the EDX analysis.

Nanoparticles can be broadly classified into two categories based on their elemental composition: metallic and non-metallic. Among the metallic NPs, those composed of metal oxides, such as zinc oxide (ZnO)^27–29^, silver oxide (Ag_2_O)^30^, iron oxide (Fe_2_O_3_)^31^, and titanium dioxide (TiO_2_), have captured widespread interest in recent years, attributed to their photocatalytic properties. The prominent non-metallic NPs are silica oxide (SiO_2_), graphene, and carbon nanotubes (CNTs)^32,33^. The utilization of actinomycetes such as *Nocardia*, *Streptomyces*, and *Thermomonospora* species to produce nanomaterials as a green approach has rapidly increased. This could be explained by the abundance of secondary metabolites that served as reducing and capping agents for synthesized nanoparticles^34–36^. Although the promising biomedical applications of TiO_2_-NPs, their synthesis using actinomycetes is low^21^. This is the first report for utilizing *Streptomyces vinaceusdrappus* in their synthesis. The characterization of TiO_2_-NPs is an important step that should be achieved before being used in various biomedical and biotechnological sectors. In the current investigation, the microscopy technique, including TEM, revealed valuable insights into the morphology and size of the biosynthesized TiO_2_-NP. The TEM micrographs showed a well-dispersed, spherical shape with a relatively uniform size distribution in the ranges from 10 nm to 50 nm (Fig. 1A, B, and C). In recent research, marine *Streptomyces* sp. was used to form oval and spherical shapes of TiO_2_-NPs with sizes from 3.5 to 92 nm^37^. On the other hand, spherical TiO_2_-NPs were fabricated using an actinomycetes strain identified as *Streptomyces* HC1sp.with sizes in the ranges of 43–67 nm^21^. The difference in the shapes and sizes may be related to the metabolites secreted by various *Streptomyces* spp. that cap NPs after production^38^. Comparatively, a photosynthetic TiO_2_-NP derived from *Punica granatum* (pomegranate) was reported to be spherical in shape with an average size of 100–150 nm^39^. Similarly, using *Cissus rotundifolia*, the TiO_2_-NP photosynthesized was spherical and sized 100 nm^40^, another round and polydisperse TiO_2_-NP with an average size of 12–30 nm synthesized from *Syzygium cumini* (Java plum)^41^ and a rectangular one with 31–42 nm was fabricated by *Tulbhagia violacea*^42^. A spherical-shaped Ag-TiO_2_NP with a size range between 25 and 50 nm was synthesized using *Origanum majorana*^43^. Further, *Zanthoxylum armatum* facilitated the synthesis of a spherical Zn-TiO_2_NP measuring 15 nm in diameter^44^.

The biosynthesis of NPs is regulated by key parameters like pH and temperature. The previous investigation reported that the nanoparticles exhibit irregular shapes and tend to aggregate at lower pH, while it promotes well-dispersed clusters at alkaline conditions^45^. Also, temperature plays a dual function where higher temperatures accelerate reduction rates, leading to smaller, highly dispersed NPs by favoring nuclei formation over secondary surface reduction. Additionally, the shape of nanoparticles differs with reactant concentration^46^. Both reaction duration and microbial levels significantly affect the synthesis process, where longer times yield larger particles through continued metal ion reduction, while higher microbial amounts speed up synthesis by providing more biological reducing agents^47^. Altogether, these factors characterize the final features of the nanoparticles.

The surface morphology and elemental contents of the prepared sample were detected by SEM-EDX analysis. As shown, the spherical particles were arranged with small aggregation (Fig. 1D and E). The presence of peaks at bending energies of 0.5 KeV (corresponding to O ion) and 0.45 and 4.5 KeV (corresponding to Ti) confirmed the formation of TiO (Fig. 1F). This finding is compatible with published investigations about the peaks of TiO_2_-NPs at specific bending energy^37,48^. The EDX analysis confirms the elemental composition, showing the presence of titanium (Ti: 4.2 wt%, 1.5 at%) and oxygen (O: 95.8 wt%, 98.5 at%), validating the main components of the prepared sample are Ti and O (Fig. 1D). The presence of other minor peaks or second peaks in EDX chart could be related to some impurities from actinobacterial metabolites that coating the synthesized NPs^49^. The difference in the percentages of atoms during EDX analysis could be related to distinct factors such as surface roughness, homogeneity of the sample during grid preparation, interaction of electron beam with the prepared sample, organic residue from capping agents, moisture content, and sensitivity of the EDX detector. However, the presence of target elements, which here are Ti and O, confirmed the successful formation of TiO_2_-NPs.

**Fig. 2:**
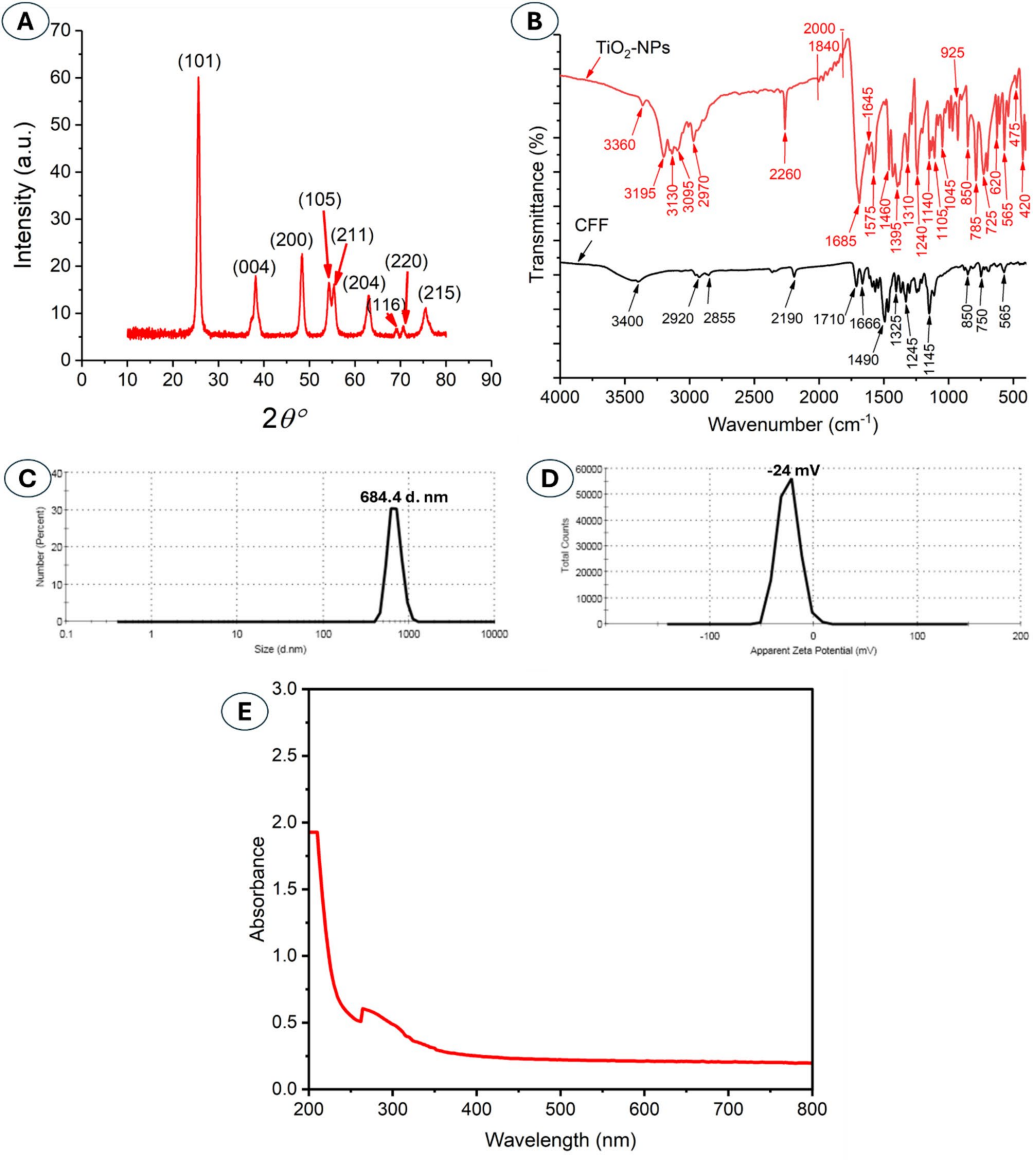
XRD (**A**), FT-IR (**B**), DLS (**C**), zeta potential (**D**), and UV–Vis spectroscopic analysis (**E**) of biosynthesized TiO_2_-NPs showing crystallinity, different functional groups, sizes in the colloidal solution, stability, and maximum SPR, respectively.

The XRD pattern exhibits distinct diffraction peaks at 2θ values of 25.57°, 38.21°, 48.25°, 54.42°, 55.45°, 63.16°, 68.98°, 70.74°, and 75.53°, corresponding to the (101), (004), (200), (105), (211), (204), (116), (220), and (215) planes, respectively (Fig. 2A), of the anatase phase of TiO_2_ (JCPDS card no. 21-1272). The high intensity and sharpness of these peaks indicate the high crystallinity of the nanoparticles and the formation of a single-phase anatase TiO_2_ without significant impurities. The XRD chart shows the presence of the highest plane (101) at 2θ values in the ranges of 24–26°, indicating the structure formed was anatase crystalline^50^. The obtained results were matched with published ones about the formation of anatase crystallographic TiO_2_-NPs^37,51^. The average size of formed crystallite TiO_2_ was assessed using the Debye-Scherrer equation based on all XRD peaks, which was 33.3 nm (Table S1, see supplementary data). As shown, the size obtained by XRD is smaller than those obtained by TEM. This finding is because the XRD measures the size of each crystallite, whereas the TEM measures the size of entire particles. Also, the size obtained by TEM represents the average total particle size, while XRD gives the average size of crystalline domains^52^.

The FT-IR spectrum of actinobacterial cell-free filtrate (CFF) exhibited characteristic peaks represented to specific groups related by different metabolites, such as carbohydrates, proteins, amino acids, polysaccharides, and amines (primary and secondary), having a vital role in NPs biosynthesis through reduction of the metal precursor. As shown, the strong and broad peak at 3400 cm^–1^ in CFF refers to the stretching N–H of primary amines overlapping with the O–H group^53,54^. This peak was shifted to more than ones, 3360, 3195, 3130, and 3095 cm^–1^ upon TiO_2_-NPs formation (Fig. 2B). Moreover, the medium peaks at 2920 and 2855 cm^–1^ in CFF indicate the presence of stretching C–H of aliphatic hydrocarbon. These peaks were shifted to 2970 cm^–1^ after TiO_2_ formation. The weak peak at 2190 cm^–1^ in CFF signifies the stretching C ≡ C of alkyne. The strong peak at 2260 cm^–1^ indicates the N = C = O of isocyanate. The appearance of medium peaks in the ranges of 1800–2000 cm^–1^ upon TiO_2_ formation indicates aromatic compounds’ bending C–H. The FT-IR analysis exhibited the formation of new peaks or changes in the intensity of peaks at the ranges of 1600–900 cm^–1^ after the formation of NPs compared to the control (CFF), which indicates the roles of lipids, proteins, and primary alcohol in the fabrication process^55^. The presence of strong peaks in the ranges of 700–400 cm^–1^ upon NPs formation refers to the successful O–Ti–O formation^37^. Derived from the FT-IR analysis, the presence of various functional groups has a main function in the reduction followed by capping and subsequently increased stabilization of biosynthesis NPs, leading to a decrease or prevention of agglomeration.

The behaviors and sizes of TiO_2_-NPs in the fluids were detected using dynamic light scattering (DLS). The data exhibit that the size of TiO_2_-NPs in the solution was 684.4 d. nm (hydrodynamic diameter) (Fig. 2C), which is bigger than the sizes obtained from TEM and XRD. This finding could be related to the DLS measure hydrodynamic diameter which means the effective size in the solution. Also, NPs’ core and any coating substances, such as proteins, will interfere and increase the obtained DLS sizes. On the other hand, the TEM analysis measures the physical size (in solid state) of the NPs’ core, whereas DLS measures sizes, including any surface coating. This is the reason for the increased sizes obtained by DLS compared to other techniques^56^. Moreover, the stability of TiO_2_-NPs was investigated by the zeta potential value that detects the surface charge. The presence of one charge on the NP’s surface (positive or negative) indicates that the repulsion between particles will increase, which decreases the agglomeration and increases stability. Also, the stability of NPs could be caused by the reducing agents, such as proteins, carbohydrates, and amines, that coat the NPs and increase their stability^4,57^. In the current study, the synthesized TiO_2_-NPs have a negative charge and an electrical potential of -24 mV (Fig. 2D).

Finally, the optical features of biogenic TiO_2_-NPs using actinobacterium strain *S. vinaceusdrappus* were assessed by UV-Vis spectroscopic analysis. Data showed that the maximum surface plasmon resonance (SPR) of synthesized titania was observed at a wavelength of 265 nm (Fig. 2E). The current result was compatible with those reported that the strong absorption peak of titania fabricated by the green method was localized at wavelengths of 240–400 nm^5^. Srinivasan and coauthors reported that the maximum SPR of titania formed by water extract of *Sesbania grandiflora* was observed at 240 nm^58^. Also, TiO_2_-NPs formed by plant extract showed two absorption peaks at a wavenumber of 245–265 nm, which is identical to titania nanostructure^59^.

### Biomedical applications

#### Antioxidant

**Fig. 3:**
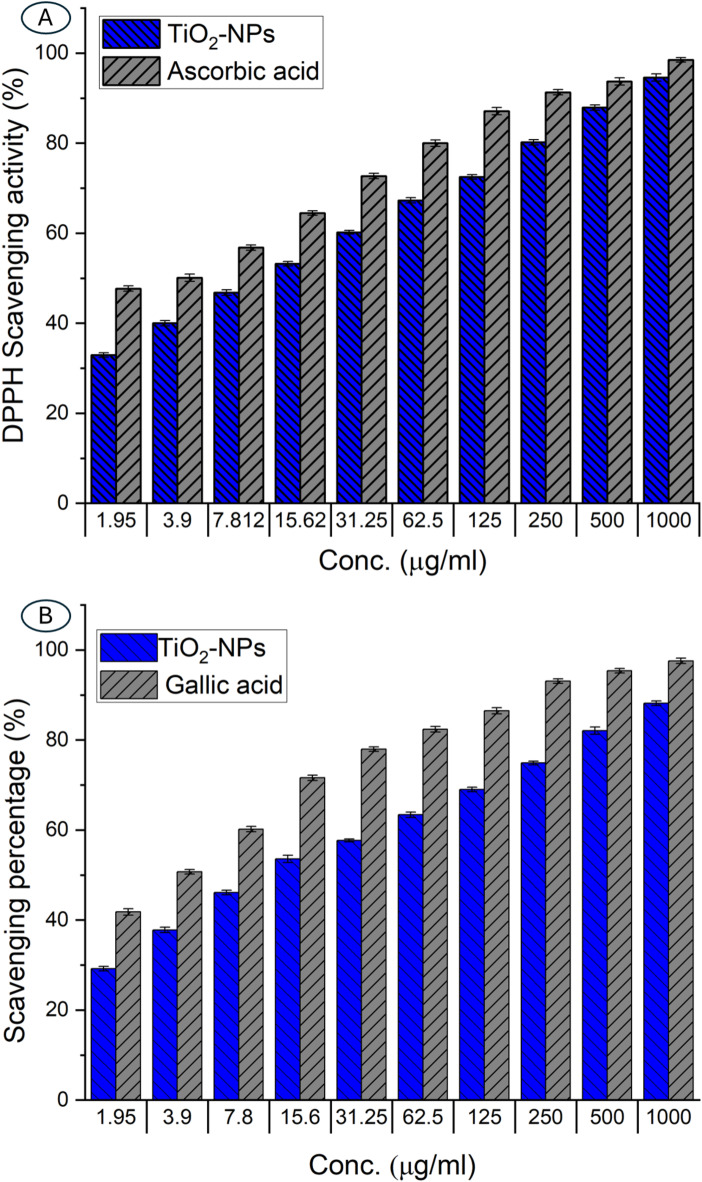
Antioxidant activity of actinobacterial mediated biosynthesis of TiO_2_-NPs. (**A**) DPPH scavenging activity compared to a positive control (ascorbic acid), and (**B**) ABTS scavenging activity of TiO_2_-NPs compared to gallic acid as a positive control. Results represented as mean ± SD. One-way ANOVA (*n* = 3, *P* ≤ 0.05).

The antioxidant activity of TiO_2_-NP was evaluated through different assay methods, including DPPH, ABTS, TAC, and FRAP. The DPPH scavenging activity was estimated at 1000–1.9 µg/ml concentrations compared to positive control (ascorbic acid). Data analysis revealed that the scavenging activity was concentration-dependent; it increased from 40% at a concentration of 3.9 µg/ml to 46.8% and 53.2% at 7.8 µg/ml and 15.6 µg/ml, respectively. Further increases in TiO_2_-NP concentrations resulted in higher DPPH scavenging percentages, reaching 60.2, 67.3, 72.5, 80.2, and 87.9% compared to the scavenging activity of ascorbic acid (72.7, 80.0, 87.1, 91.3, and 93.7%) at concentrations of 31.2, 62.5, 125, 250, and 500 µg/ml respectively (Fig. 3A). The highest tested concentration of 1000 µg/ml TiO_2_-NP exhibited a considerable 94.6% DPPH scavenging activity compared to a percentage of 98.5% for ascorbic acid. In a similar investigation, green synthesized TiO_2_-NPs showed varied DPPH scavenging activity based on the concentration used^60^. The authors noted that the maximum scavenging percentage was attained at a concentration of 500 µg/ml, and the activity decreased to the lowest at 10 µg/ml. The authors concluded that the DPPH scavenging activity depended on the concentration, shape, and size of TiO_2_-NPs. The detection of IC_50_ value (concentration that scavenges 50% of free radicals) is a pivotal factor that should be detected before using NPs in biomedical sectors. In the current investigation, the IC_50_ value of actinobacterial synthesized TiO_2_-NPs was 11.1 µg/ml compared to 2.85 µg/ml of ascorbic acid.

In the ABTS.^**+**^ radical scavenging assay, data analysis shows a dose-dependent increase in the scavenging activity, indicating a direct correlation between the concentration of TiO_2_-NP and its ability to neutralize ABTS radicals. At the lowest concentration of 1.9 µg/ml, TiO_2_-NP exhibited modest 29.2% scavenging activity, gradually increasing to 37.8, 46.1, 53.6, and 57.7% as the concentration doubled from 3.9 to 31.2 µg/ml. A more significant jump in activity was observed at higher concentrations, reaching 63.4%, 69.0%, 74.9%, 82.1%, and a maximum of 88.2% at 1000 µg/ml compared to scavenging percent of 97.6% of gallic acid at the same concentration (Fig. 3B). The IC_50_ value was 14.36 µg/ml for TiO_2_-NP, while gallic acid exhibited an IC_50_ of 2.54 µg/ml. Similarly, the IC_50_ value of TiO_2_-NPs formed by green tea as a reducing agent was 19.6 µg/ml compared to 16.8 µg/ml for positive control and 33.5 µg/ml compared to 29.5 µg/ml for positive control for DPPH and ABTS assay methods respectively^61^.

Complementing these findings, the TAC assay revealed a mean value of 505.4 ± 3.3 µg/mg antioxidant activity equivalent (AAE), while the FRAP assay yielded a mean value of 395.3 ± 8.1 µg/mg AAE for TiO_2_-NP. These results indicate that TiO_2_-NP possesses a significant overall antioxidant capacity, as demonstrated by their capacity to neutralize free radicals, reduce ferric ions, and exhibit total antioxidant capacity. The antioxidant activity of green-formed NPs varied based on the metabolites or phytoconstituents that act as capping and stabilizing agents. For instance, the DPPH scavenging activity of TiO_2_-NPs formed by aqueous extract of plum peels is higher compared to those formed by peach and kiwi due to the high plum peel metabolites^62^.

### Wound healing

**Fig. 4:**
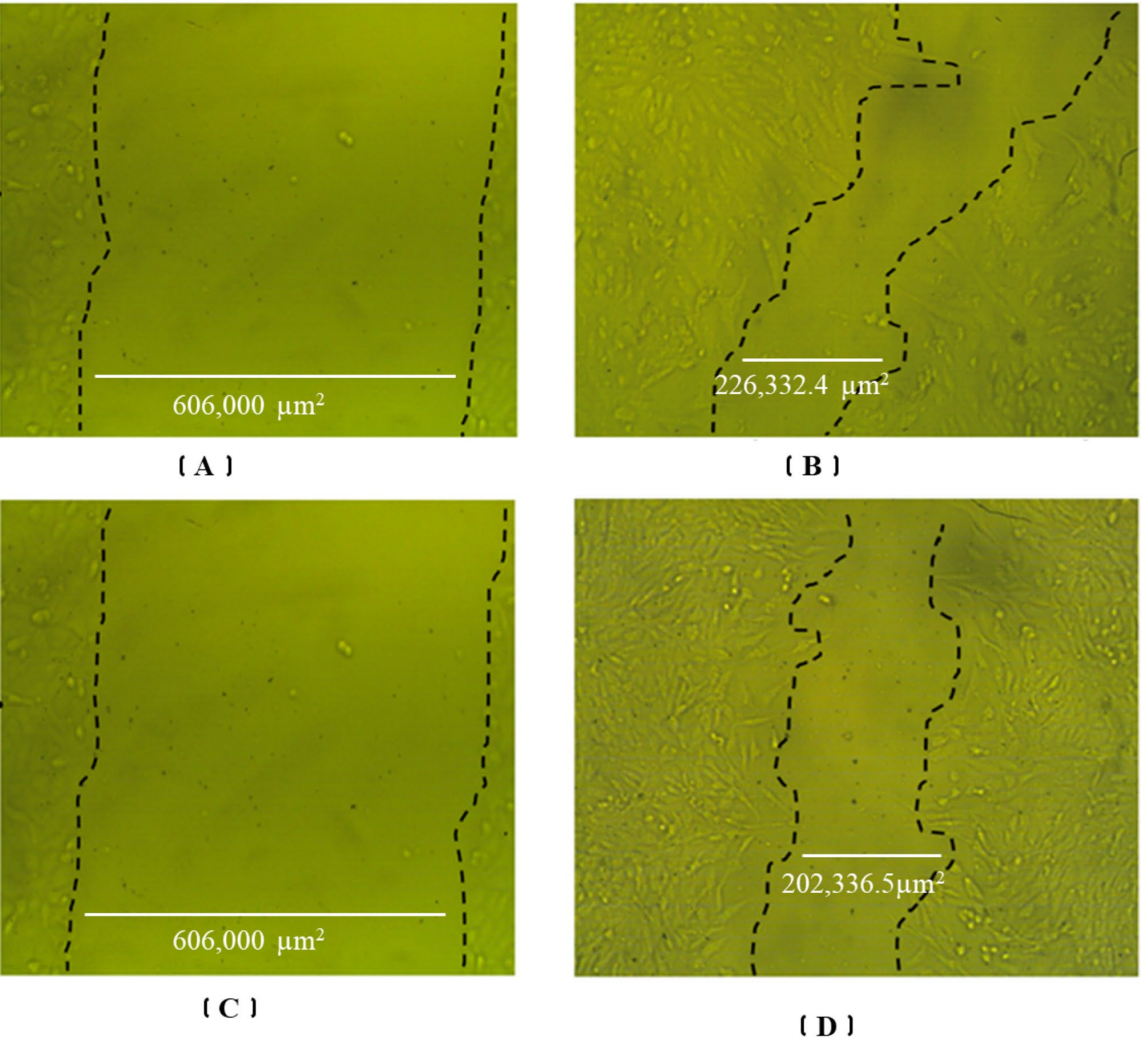
Wound healing assay comparing the effects of TiO_2_-NP treatment (76.54 µg) and control on wound closure in vitro. Representative images show the wound area at 0 h (initial) and 48 h after treatment. (**A**) Untreated cells at 0 h (**B**) Treated cells with control after 48 h (**C**) Untreated cells at 0 h (**D**) Treated cells with TiO_2_-NP after 48 h.

TiO_2_-NP was investigated as a wound-healing agent by evaluating its effect on wound closure compared to a control group. The accompanying figure visually represents the wound healing process in the control and TiO_2_-NP treated groups (76.54 µg). It depicts the wound area at the initial time point (0 h) and after 48 h of treatment, clearly illustrating the enhanced wound closure effect of TiO_2_-NP as the wound area is visibly smaller and more contracted in the treated group compared to the control after 48 h (Table 2; Fig. 4). Data analysis showed that the wound healing potential of TiO_2_-NP compared to a control group, at the initial time point (0 h), the mean wound width and area were similar for both groups, around 794 μm and 606,000 µm^2^, respectively. After 48 h, the control group exhibited a mean wound width of 296.75 μm and an area of 226,332.4 µm^2^, indicating partial wound closure (Table 2; Fig. 4). Notably, the TiO_2_-NP treated group showed a smaller mean wound width of 265.11 μm and a reduced area of 202,336.5 µm^2^, suggesting enhanced wound closure compared to the control. The mean wound closure percentage was 62.6% for the control and 66.6% for the TiO_2_-NP group, further confirming the improved wound healing effect of the nanoparticles. The area difference highlights the quantitative difference in wound area between the two groups at 48 h, with the TiO_2_-NP group having a smaller remaining wound area of 403,457.4 µm^2^.

**Table 2:** Quantitative analysis of wound healing in control and TiO_2_-NP treated groups at 0 and 48 h. Data represents mean values of wound width, wound area, wound closure percentage, and area difference between groups at 48 h. Data represented as the means of six replicate.

Treatment	At 0 h		At 48 h		RM (µm/h)	Wound closure (%)	Area difference (µm^2^)
	Width (µm)	Area (µm²)	Width (µm)	Area (µm²)			
Control cells	794.4	605793.9	296.8	226332.4	10.4	62.6	379461.5
TiO_2_-NPs	794.4	605793.9	265.1	202336.5	11.2	66.6	403457.4

Wounds are categorized as acute or chronic based on healing time and complications^63^. The wound healing process involves four interrelated phases: hemostasis, inflammation, proliferation, and maturation, requiring coordination between various cell types, coagulation and growth factors, and the vascular system. Factors like diabetes, obesity, malnutrition, medications, and unhealthy lifestyles can impair healing^64^. Current therapies include hyperbaric oxygen, negative pressure therapy, bioengineered constructs, dressings, surgery, and medications like steroids, non-steroidal anti-inflammatory drugs, and chemotherapeutic agents, though these drugs have side effects limiting their use^65,66^. It is reported that inorganic metallic nanoparticles, such as TiO_2_-NPs, induce ROS production and activate the angiogenesis process, which is the formation of new blood vessels. This occurs through the downregulation of the p38MAPK/Akt/eNOS-dependent pathway and the upregulation of key angiogenic growth factors like vascular endothelial growth factor (VEGF) and fibroblast growth factor (FGF), ultimately accelerating wound healing^67,68^. Understanding the molecular mechanisms of inorganic metallic nanoparticles in wound healing is crucial to developing them as alternative wound treatments. Extensive research on their effects in different wound healing phases, toxicity, and biocompatibility is necessary to establish their therapeutic potential.

### Hemocompatibility assessment

Anticoagulant activity of TiO_2_-NPs was examined using prothrombin time (PT) and partial thromboplastin time (PTT) assays, which tested nanoparticle concentrations of 0, 25, 50, and 75 µg/ml. In the PT test, at 0 µg/ml concentration, TiO_2_-NPs showed almost no net change in clotting time (12.5 s), which was almost identical to the control heparin (12.0 s). With increasing TiO_2_-NPs concentration to 75 µg/ml, the time of clotting was only slightly increased (14.2 s), showing a very low dose-dependent anticoagulant effect. The PTT results demonstrated that TiO_2_-NPs had a dose-dependent increase in anticoagulant activity as the level of clotting was prolonged, increasing from 25 s at 0 µg/ml to 43 s at 75 µg/ml concentration (Table 3). Meanwhile, the heparin control exhibited significant variations in clotting time (ranging from 28 to 277 s).

**Table 3:** Assessment of concentration and clotting time measurements of ( PT and PTT) time changes induced by TiO_2_-NPs.

Conc.(µg/ml)	PT (s)—TiO_2_-NP	PT (s)—Heparin	PTT (s)—TiO_2_-NP	PTT (s)—Heparin
0	12.5	12.0	25	28
25	12.9	100	27	123
50	13.1	175	30	202
75	14.2	243	43	277

As a follow up, the hemocompatibility of TiO_2_-NPs was subjected to thorough analysis in comparison to the control conditions. The complete hemolysis control, achieved with the deionized water, showed consistent OD readings at 0.987, 1.032, and 1.002, averaging 1.007 to represent 100% hemolysis (Table 4). The hemolytic activity of TiO_2_-NPs was quite negligible, as the highest concentration of 1000 µg/ml showed only 1.9% hemolysis. Decreasing concentration also showed decreased minimal hemolytic activity: 800 µg/ml yielded 1.6% hemolysis, 600 µg/ml presented 0.7%, 400 µg/ml showed 0.3%, and 200 µg/ml indicated 0.2% hemolysis (Table 4; Fig.S4). At the lowest tested concentrations of 25 and 50 µg/mL, hemolytic activity was absent, where TiO_2_-NPs displayed 0.1% and 0.0% hemolysis, respectively, corresponding to the isotonic solution background. The TiO_2_-NPs did not exhibit any significant lysis of RBCs, thus determining their hemocompatibility for most blood-contact biomedical applications.

**Table 4:** Evaluation of hemolytic activities of TiO_2_-NPs at various concentrations.

TiO_2_-NPs concentration (µg/ml)	Mean absorbance ± SD (TiO_2_-NP with RBCs)	TiO_2_-NP with isotonic solution	Hemolysis (%)
Complete hemolysis (control)	1.007 ± 0.023	1.007	100
1000	0.040 ± 0.005	0.021	1.9
800	0.027 ± 0.005	0.011	1.6
600	0.016 ± 0.003	0.009	0.7
400	0.008 ± 0.001	0.005	0.3
200	0.005 ± 0.002	0.003	0.2
100	0.002 ± 0.001	0.001	0.1
50	0.001 ± 0.002	0.000	0
25	0.001 ± 0.001	0.000	0

#### In-vitro cytotoxicity

Next, the cytotoxicity of TiO_2_-NP was evaluated against the WI38 normal cell line to determine the safe dosage range and against Caco-2 and PANC-1 cancer cell lines to assess its potential as an anti-cancer agent. The morphological assessment revealed slight changes in the treated cells, unlike the untreated control. In the control group, the cells exhibited a typical spread-out, flattened morphology characteristic of adherent cell lines, with an even distribution across the surface and a distinct cytoplasmic area surrounding the nucleus, indicating a healthy and actively proliferating state (Fig. S1, see supplementary data). On the other hand, the cells treated with TiO_2_-NP displayed alterations in their morphology, particularly at higher concentrations, but at lower doses (31.25 and 62.5 µg/ml), the cells maintained a relatively normal appearance, resembling the control cells.

**Fig. 5:**
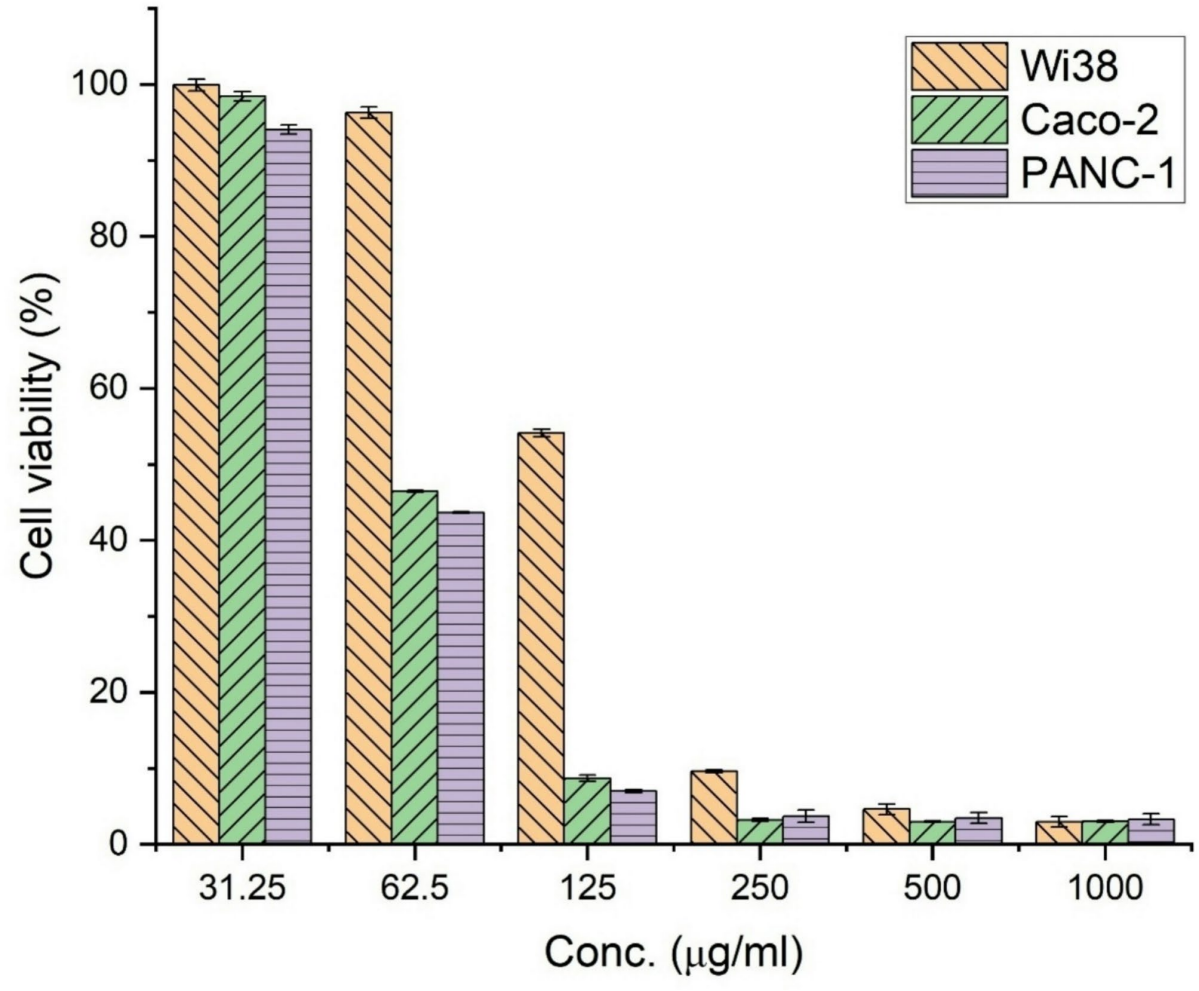
Comparative analysis of cell viability of Wi38, Caco-2, and PANC-1 cells exposed to variable concentrations of actinobacterial synthesized TiO_2_-NPs.

The quantitative cytotoxicity data corroborated the morphological observations. The viability of the untreated control cells (Wi38) was set at 100%, serving as the baseline for comparison. At the highest 1000 µg/ml concentration, TiO_2_-NP exhibited 2.95% cell viability remaining for normal cell line (Wi38). As the concentration decreased, viability gradually increased to 54.15% at 125 µg/ml. Interestingly, at lower concentrations of 62.5 µg/ml and 31.25 µg/ml, the viability remained relatively high, at 96.30% and 99.91%, respectively, suggesting minimal cytotoxic effects. The IC_50_ value of TiO_2_-NPs against the normal cell line was reported as 153.1 ± 1.01 µg/ml (Fig. 5).

For the Caco-2 cancer cell line, the morphological assessment revealed distinct changes in the treated cancer cells in contrast to the untreated control (Fig. S2, see supplementary data). In the control group, the untreated Caco-2 cells exhibited a typical epithelial-like morphology, appearing as a monolayer of adherent cells with a cobblestone or polygonal shape. The cells were evenly distributed across the surface, indicating a healthy and proliferating state. However, the cells treated with TiO_2_-NP displayed significant morphological alterations, particularly at higher concentrations. At lower doses (31.25 µg/ml), the cells maintained a relatively normal appearance, similar to the control. As the concentration increased (62.5 µg/ml), some cells appeared rounded and detached from the surface, suggesting the onset of cytotoxic effects. The morphological changes became more pronounced at higher concentrations (125, 250, 500, and 1000 µg/ml). The cells exhibited a marked loss of their characteristic epithelial-like morphology, becoming rounded, shrunken, and detached from the surface. Additionally, the cell density decreased substantially, indicating a reduction in cell viability and proliferation rates (Figure S2, see supplementary data). These morphological alterations, including cell rounding, detachment, and diminished cell density, indicate cytotoxic effects and compromised cellular functions in response to the increasing concentrations of TiO_2_ nanoparticles.

The quantitative cytotoxicity data supported morphological observations. The viability of the untreated Caco-2 (control) cells was set at 100%. At the highest 1000 µg/ml concentration, TiO_2_-NP exhibited severe cytotoxicity, with only 2.99% cell viability remaining. As the concentration decreased, viability gradually increased, reaching 46.43% at 62.5 µg/ml and 98.46% at 31.25 µg/ml (Fig. 5). Its IC_50_ value of TiO_2_-NPs against Caco-2 cancer cells was 74.14 ± 0.65 µg/ml, indicating significant cytotoxicity at concentrations above this value.

Concerning the PANC-1 pancreatic cancer cell line, the untreated cells exhibited a typical epithelial-like morphology, appearing as a monolayer of adherent cells with a cobblestone or polygonal shape. However, the cells treated with TiO_2_-NP displayed significant morphological alterations, particularly at higher concentrations. At lower doses (31.25 µg/ml), the cells maintained a relatively normal appearance, similar to the control, with some cells starting to show signs of rounding and detachment. As the concentration increased (62.5 µg/ml), more cells became rounded and detached from the surface, suggesting the onset of cytotoxic effects. The morphological changes became increasingly severe at higher concentrations (125, 250, 500, and 1000 µg/ml). The cells exhibited a marked loss of their characteristic epithelial-like morphology, appearing highly rounded, shrunken, and completely detached from the surface. Additionally, the cell density decreased substantially, with large areas devoid of cells, indicating a significant reduction in cell viability and proliferation rates. These morphological alterations, including cell rounding, shrinkage, detachment, and diminished cell density, indicate severe cytotoxic effects and compromised cellular functions in response to the increasing concentrations of TiO_2_ nanoparticles (Fig. S3, see supplementary data). Furthermore, the treated cells displayed signs of membrane blebbing, a characteristic feature of apoptosis or programmed cell death. The presence of membrane blebs, which are small protrusions or bulges on the cell surface, suggests that TiO_2_-NP treatment may have induced apoptotic pathways in the PANC-1 cells, leading to their eventual demise.

The quantitative cytotoxicity data supported morphological observations. The viability of the untreated control cells was set at 100%. At the highest 1000 µg/ml concentration, TiO_2_-NP exhibited severe cytotoxicity, with only 3.28% cell viability remaining. As the concentration decreased, viability gradually increased, reaching 43.65% at 62.5 µg/ml and 94.04% at 31.25 µg/ml (Fig. 5). The IC_50_ value against PANC-1 was 71.04 ± 1.17 µg/ml.

TiO_2_ is photoactive, which induces reactive oxygen species when subjected to light. These ROS can induce selective cell death in cancer cells^69^. Furthermore, the production of ROS by TiO_2_-NPs has been reported to act as effector signaling mediators in the p53-dependent apoptotic pathway. Upregulated expression of cytochrome c, cleaved caspase-3, and Poly (ADP-ribose) polymerase (PARP) indicated the induction of apoptosis via caspase activation, highlighting the therapeutic promise of surface-engineered TiO_2_-NPs^23,70^. Additionally, the generated ROS can damage the mitochondrial membrane and its functionality, subsequently initiating the mitochondrial release of cytochrome c into the cellular matrix, thereby triggering the intrinsic apoptotic cascade^71,72^. Moreover, the proliferation and survival of cancer cells can be affected by the interaction of NPs with signaling pathways, leading to the dysfunction of proteins and enzymes needed for cellular processes^73^.

#### Antidiabetic activity

**Fig. 6:**
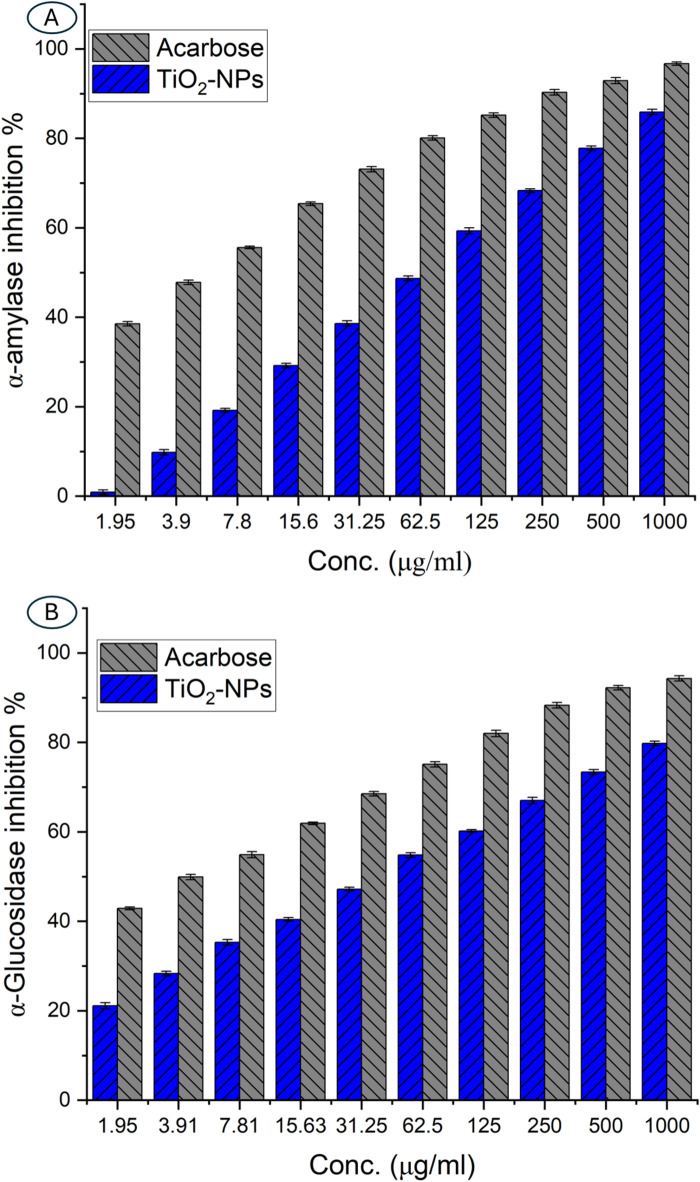
Comparative analysis of in-vitro inhibition % of α-Amylase and α-Glucosidase by TiO_2_-NP and Acarbose at conc. (1.9–1000 µg/ml).

The antidiabetic potential of TiO_2_-NP was evaluated by assessing its inhibitory effects on the carbohydrate-hydrolyzing enzyme α-amylase. The results demonstrate a concentration-dependent inhibition of α-amylase by TiO_2_-NP, with inhibition percentages ranging from 0.9% at the lowest tested concentration of 1.9 µg/ml to 85.9% at the highest concentration of 1000 µg/ml. The IC_50_ value of TiO_2_-NP against α-amylase was found to be 69.3 µg/ml, while the standard α-amylase inhibitor acarbose exhibited an IC_50_ of 4.03 µg/ml. Notably, the inhibitory activity showed a substantial increase as the concentration increased, with inhibition percentages of 19.2%, 29.2%, and 38.6% observed at concentrations of 7.8, 15.6, and 31.2 µg/ml, respectively. Higher concentrations of 62.5, 125, 250, and 500 µg/ml resulted in inhibition percentages of 48.7%, 59.3%, 68.3%, and 77.8%, respectively (Fig. 6A).

Furthermore, TiO_2_-NP also exhibited potent inhibitory effects on the enzyme α-glucosidase; at the lowest concentration of 1.9 µg/ml, TiO_2_-NPs demonstrated a 21.1% inhibition of α-glucosidase, which increased to 28.3, 35.3, 40.4, and 47.2% at concentrations of 3.9, 7.8, 15.6, and 31.2 µg/ml, respectively. The IC_50_ value of TiO_2_-NPs against α-glucosidase was found to be 40.81 µg/ml, while the standard α-glucosidase inhibitor acarbose exhibited an IC_50_ of 3.92 µg/ml. Higher concentrations of 62.5, 125, 250, and 500 µg/ml resulted in α-glucosidase inhibition percentages of 54.8, 60.2, 67.0, and 73.4%, respectively, with the highest tested concentration of 1000 µg/ml exhibiting 79.8% inhibition compared to 94.3% of acarbose at the same concentration (Fig. 6B).

Recently, green synthesized TiO_2_-NPs showed promising antidiabetic activity via inhibition of hydrolytic α-amylase and α-glucosidase enzymes, which are useful in type-2 diabetes treatment by decreasing hyperglycemia^74^. In a recent investigation, the inhibitions of hydrolytic α-amylase and α-glucosidase were concentrations TiO_2_-NPs dependent^75^. The authors reported that the α-amylase enzyme was inhibited with percentages of 17% at a concentration of 100 µg/ml with IC_50_ value of 46.2 µg/ml compared to 39% inhibition percentages caused by acarbose with IC_50_ value of 21.4 µg/ml. Whereas the α-glucosidase enzyme was inhibited with percentages of 37% at 100 µg/ml with an IC_50_ value of 31.3 µg/ml. In the current investigation, the inhibition percentages of α-glucosidase at low concentrations (1.9–125 µg/ml) were better than the inhibition of α-amylase enzyme. The importance of this finding is that α-glucosidase has major effects on sugar metabolism compared to α-amylase^76^. Therefore, it can be concluded that the actinobacterial synthesized TiO_2_-NPs can be considered a promising antidiabetic agent at low concentrations. The antioxidant activity of TiO_2_-NPs (as mentioned above) helps to decrease oxidative stress, which is regarded as the main reason for infection by diabetes disease, especially type-2^77^. Also, the pancreatic β-cells, which are responsible for the production and secretion of insulin, can be protected by TiO_2_-NPs by decreasing the oxidative stresses, leading to glucose regulation in the blood^78^. TiO_2_-NPs can stimulate glucose utilization by enhancing the glucose transporter protein expression, improving glucose uptake by muscles and fat cells.

#### Antimicrobial activity

**Fig. 7:**
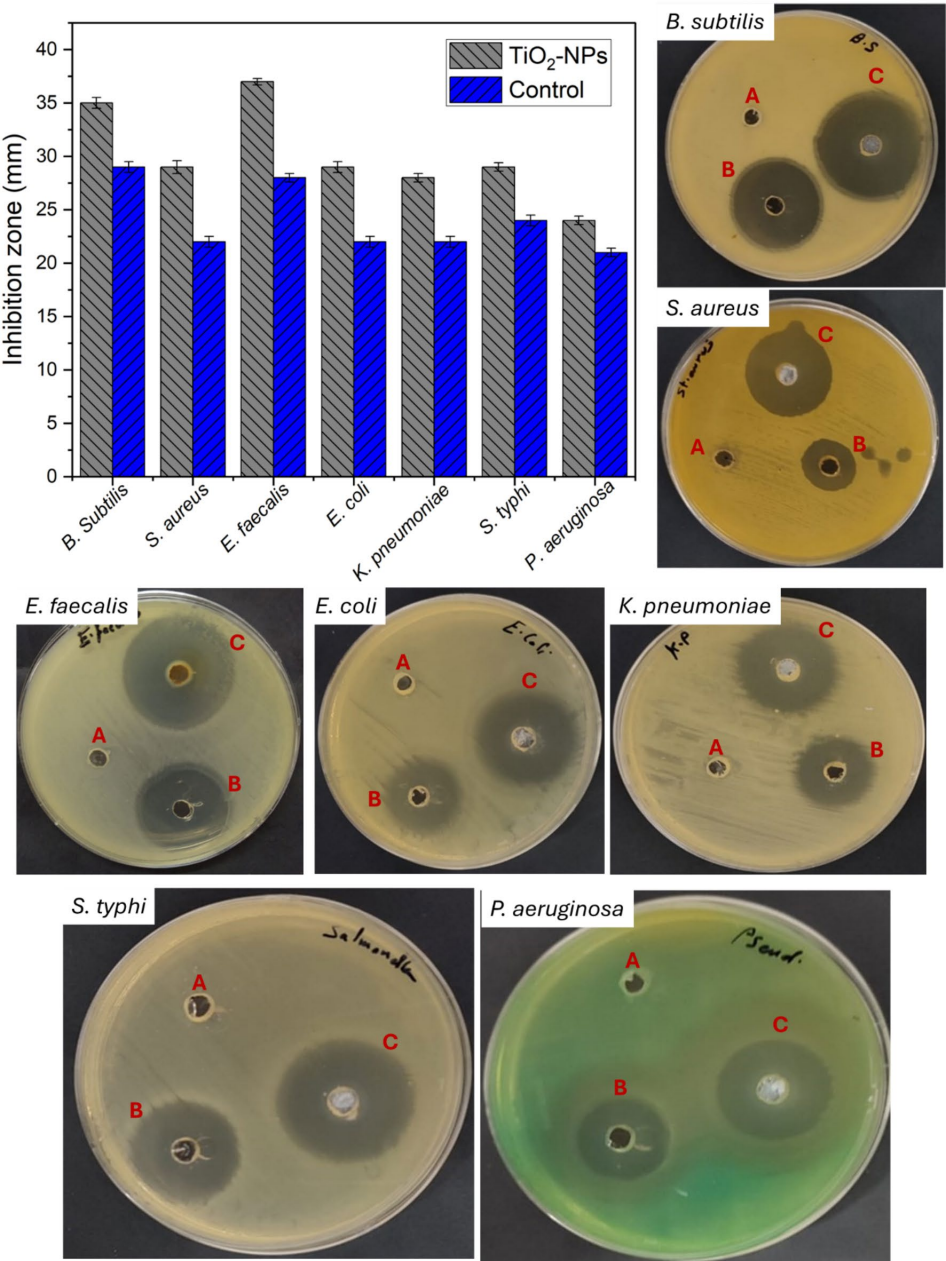
Antibacterial activity of TiO_2_-NPs against different pathogenic bacterial strains showing data analysis and clear zones formed due to treatment with 1 mg/ml of synthesized NPs compared to positive and negative control. (**A**) the negative control (DMSO), (**B**) the positive control (gentamicin), and (**C**) the TiO_2_-NPs.

**Fig. 8:**
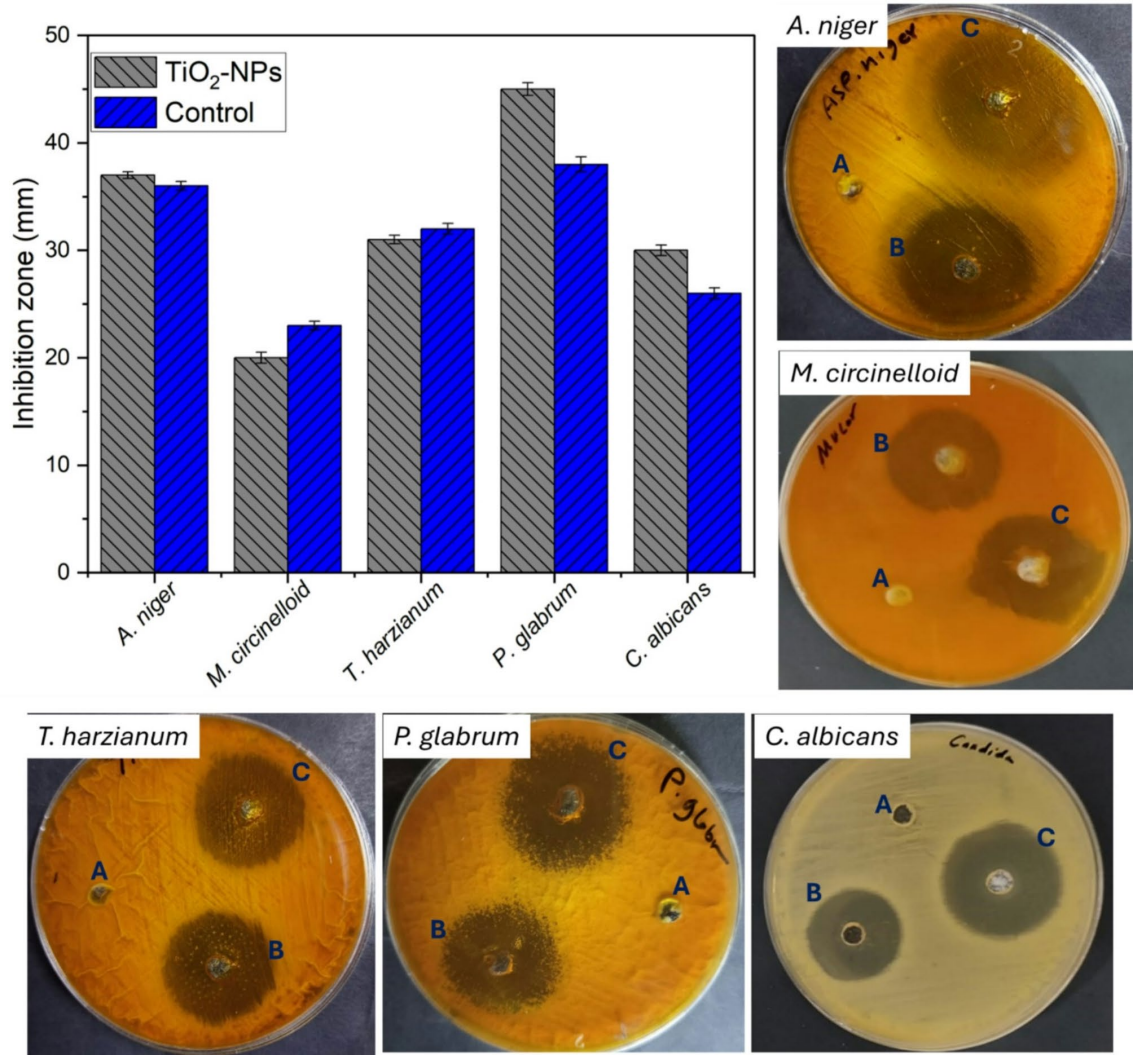
Antifungal activity of actinobacterial-TiO_2_-NPs against various multi- and unicellular fungal strains showing data analysis and clear zones formed upon treatment with a concentration of 1 mg/ml TiO_2_-NPs. (**A**) the negative control (DMSO), (**B**) the positive control (fluconazole), and (**C**) is the TiO_2_-NPs.

The actinobacterial-mediated biosynthesis of TiO_2_-NPs exhibits varied activity against pathogenic Gram-positive (G+) and Gram-negative (G-) bacterial strains and eukaryotic species, including multicellular and unicellular fungi. TiO_2_-NP displayed promising antibacterial activity against the three G + bacteria tested, *Bacillus subtilis*, *Staphylococcus aureus*, and *Enterococcus faecalis*, as evidenced by the larger inhibition zones compared to the control gentamicin, suggesting its stronger antibacterial potency (Fig. 7). Notably, TiO_2_-NP displayed inhibition zones of 35 ± 0.1, 29 ± 0.2, and 37 ± 0.1 mm against *B. subtilis*, *S. aureus*, and *E. faecalis*, respectively, whereas gentamicin exhibited inhibition zones of 29 ± 0.1, 22 ± 0.3, and 28 ± 0.1 mm, respectively. Concerning the G-ve bacteria, TiO_2_-NP exhibited varying antibacterial activity against the tested G-ve bacteria, *Escherichia coli*, *Klebsiella pneumoniae*, *Salmonella typhi*, and *Pseudomonas aeruginosa* (Fig. 8). Against *E. coli*, TiO_2_-NP displayed a larger inhibition zone (29 ± 0.1 mm) compared to gentamicin (22 ± 0.2 mm), suggesting stronger antibacterial potency. Moreover, for *K. pneumoniae*, *S. typhi*, and *P. aeruginosa*, TiO_2_-NP exhibited slightly larger inhibition zones (28 ± 0.1, 29 ± 0.2, and 24 ± 0.2 mm, respectively) compared to gentamicin (22 ± 0.2, 24 ± 0.1, and 21 ± 0.1 mm, respectively), suggesting moderate antibacterial activity.

Interestingly, TiO_2_-NP exhibited low MIC values of 12.5 µg/ml against all three G + bacteria, indicating its effectiveness in inhibiting their growth at relatively low concentrations. Correspondingly, MBC values of 25 µg/ml against these bacteria demonstrated TiO_2_-NP potent bactericidal activity. Moreover, the MBC/MIC ratio of 2 for all three G + ve pathogens confirmed TiO_2_-NP’s ability to exhibit bactericidal activity against them (Table 5). Notably, the actinobacterial- TiO_2_-NP exhibits a lower MIC value of 6.25 µg/ml and a lower MBC value of 12.5 µg/ml against *E. coli*, indicating its effectiveness in inhibiting and killing this bacterium at relatively low concentrations. The MBC/MIC ratio 2 confirmed TiO_2_-NP’s bactericidal activity against *E. coli*. However, the MIC values for *K. pneumoniae*, *S. typhi*, and *P. aeruginosa* were higher at 12.5 µg/ml, indicating a lower potency in inhibiting their growth than *E. coli*. Furthermore, the MBC values for these bacteria were relatively high at 50 µg/ml, and the MBC/MIC ratio for these three bacteria was 4, indicating a higher concentration requirement for bactericidal activity compared to the G + ve bacteria and *E. coli* (Table 5).

**Table 5:** MIC, MBC, MFC, and their ratios (MBC/MIC and MFC/MIC) of TiO_2_-NPs against various bacterial and fungal strains.

Tested bacterial strains	MIC (µg/ml)	MBC (µg/ml)	MBC/MIC ratio	Tested filamentous fungi and yeasts	MIC (µg/ml)	MFC (µg/ml)	MFC/MIC ratio
*Bacillus Subtilis*	12.5	25	2	*Aspergillus niger*	6.25	50	8
*Staphylococcus aureus*	12.5	25	2	*Mucor circinelloides*	100	300	3
*Enterococcus faecalis*	12.5	25	2	*Trichoderma harzianum*	50	300	6
*Escherichia coli*	6.25	12.5	2	*Penicillium glabrum*	12.5	200	16
*K. pneumoniae*	12.5	50	4	*Candida albicans*	6.25	25	4
*Salmonella typhi*	12.5	50	4				
*Pseudomonas aeruginosa*	12.5	50	4				

Consequent to our findings, a 20 nm diameter smooth and spherical TiO_2_-NP was biosynthesized by *Staphylococcus aureus*, which exhibited antibacterial and antibiofilm activity against *B. subtilis* and *E. coli*^79^. Another spherical TiO_2_-NP measuring 80 nm in diameter was synthesized from *Hypsizygus ulmarius*, which demonstrated its ability to inhibit the growth of *S. aureus*, *B. cereus*, *E. coli*, and *K. pneumoniae* with inhibition zones measuring 8.4, 7.1, 6.2, and 4.0 mm, respectively^80^. Moreover, a 31 nm TiO_2_-NP bioformed in sphere shape using *Pleurotus djamor* also showed antibacterial activity against *P. fluorescens*, *S. aureus*, and *Corynebacterium diphtheriae* organisms^81^. Additionally, the mycofabricated TiO_2_-NP by *P. ostreatus* with an average size of 20–50 nm was capable of inhibiting the bacterial growth of *S. mutans* and *S. epidermidis*^82^ using the disc diffusion method.

Data analysis showed that the TiO_2_-NP has antifungal activity against several filamentous fungi and yeasts, with varying degrees of efficacy. The nano compound was particularly effective against *Penicillium glabrum*, *Aspergillus niger*, and *Candida albicans*, as indicated by larger inhibition zones and lower MIC and MFC values compared to other tested fungi (Fig. 8). However, it showed relatively weaker activity against *Mucor circinelloides*, as evidenced by a smaller inhibition zone and higher MIC value. Notably, TiO_2_-NP displayed superior antifungal potency compared to the control fluconazole against *P. glabrum*, with a larger inhibition zone (45 ± 0.1 mm vs. 38 ± 0.1 mm) and lower MIC (12.5 µg/ml) and MFC (200 µg/ml) values (Table 5). Similarly, TiO_2_-NP outperformed fluconazole in inhibiting *A. niger* growth, with a slightly larger inhibition zone (37 ± 0.2 mm vs. 36 ± 0.1 mm) and lower MIC (6.25 µg/ml) and MFC (50 µg/ml) values (Table 5). Against *C. albicans*, TiO_2_-NP exhibited comparable antifungal activity to fluconazole, with a slightly larger inhibition zone (30 ± 0.3 mm vs. 26 ± 0.3 mm) and similar MIC (6.25 µg/ml) but a lower MFC (25 µg/ml). Also, it displayed weaker antifungal potency against *M. circinelloides* compared to fluconazole (Fig. 8), with a smaller inhibition zone (20 ± 0.2 mm vs. 23 ± 0.2 mm) and higher MIC (100 µg/ml), while no MFC value was reported (Table 5).

TiO_2_-NP exhibits potent antimicrobial activity through various mechanisms. Upon interacting with microbial cells, these NPs trigger the generation of ROS, which can compromise the integrity of the cell wall by oxidizing phospholipids^18^. These produced ROS can also inflict damage on essential cytosolic enzymes and disrupt the structural integrity of macromolecules within the cell^34^, ultimately affecting cellular functions and gene expression^83^. Moreover, TiO_2_-NP can alter the ionic balance inside microbial cells, leading to reduced phosphate uptake and hindered cell-to-cell communication, further compromising the survival and proliferation of these microorganisms^84^.

The difference in efficacy against G + and G- bacteria arises from the distinct cell wall structures. The well-dispersed and spherical morphology and relatively uniform size distribution of TiO_2_-NP, observed through SEM and TEM, contribute to the antimicrobial properties. Where the negative charge, with a zeta potential of -30.6 mV, facilitates better access and penetration of the nanoparticles into the thicker peptidoglycan layer of G + bacteria, enabling ROS generation in close proximity to the cell wall and cytoplasmic membrane. In contrast, G- bacteria possess an additional outer membrane composed of lipopolysaccharides (LPS), as confirmed by the EDX analysis, which shows the presence of only titanium and oxygen elements. This outer membrane acts as a barrier, limiting the interaction and penetration of the TiO_2_-NPs, and the negatively charged LPS can electrostatically repel the negatively charged nanoparticles, reducing their antimicrobial efficacy against G- bacteria compared to G + bacteria^85^.

The biosynthesized TiO_2_-NP exhibited antifungal capabilities due to their interaction with the fungal cell wall and membrane components, as suggested by the presence of various functional groups observed in the FT-IR spectrum, indicating the involvement of biomolecules from the actinobacterium cell-free filtrate during the synthesis process. The negative charge on the nanoparticles, with a zeta potential of -30.6 mV, likely facilitates their inhibitory binding to charged cell walls and membrane components like mannoproteins, glucans, and chitin. This binding can inhibit crucial enzymes responsible for cell wall synthesis, such as β-glucan synthase. Moreover, the small size range of 10–50 nm and high surface area, revealed by TEM and XRD analyses, can enhance the nanoparticles’ surface reactivity and penetration into fungal cells, inducing the generation of ROS and subsequent oxidative stress, ultimately leading to the disruption of macromolecules like DNA, RNA, and proteins, causing cell death^86^.

In the current investigation, TiO_2_-NPs have relatively weaker antifungal activity than their antibacterial action. This difference can be attributed to the fundamental structural distinctions between the cellular envelopes of fungi and bacteria^87^. Whereas fungal cells possess a more robust and intricate cell wall architecture, bacterial cells exhibit a relatively simpler cell envelope structure^88^. For example, one study reported the synthesis of spherical-shaped titanium TiO_2_-NPs, ranging in size from 30 to 70 nm, using *Streptomyces* sp. HCl. The developed TiO_2_-NPs were evaluated for their antimicrobial activity against various pathogenic microorganisms, including *Staphylococcus aureus*, *Escherichia coli*, *Candida albicans*, and *Aspergillus niger*. The researchers concluded that the synthesized TiO_2_-NPs exhibited higher activity against bacterial species compared to fungal species^21^. Consequently, the complex composition of the fungal cell wall may confer greater resistance against the antifungal mechanisms of TiO_2_-NPs, thereby rendering fungi less susceptible compared to their bacterial counterparts^89^.

**Fig. 9:**
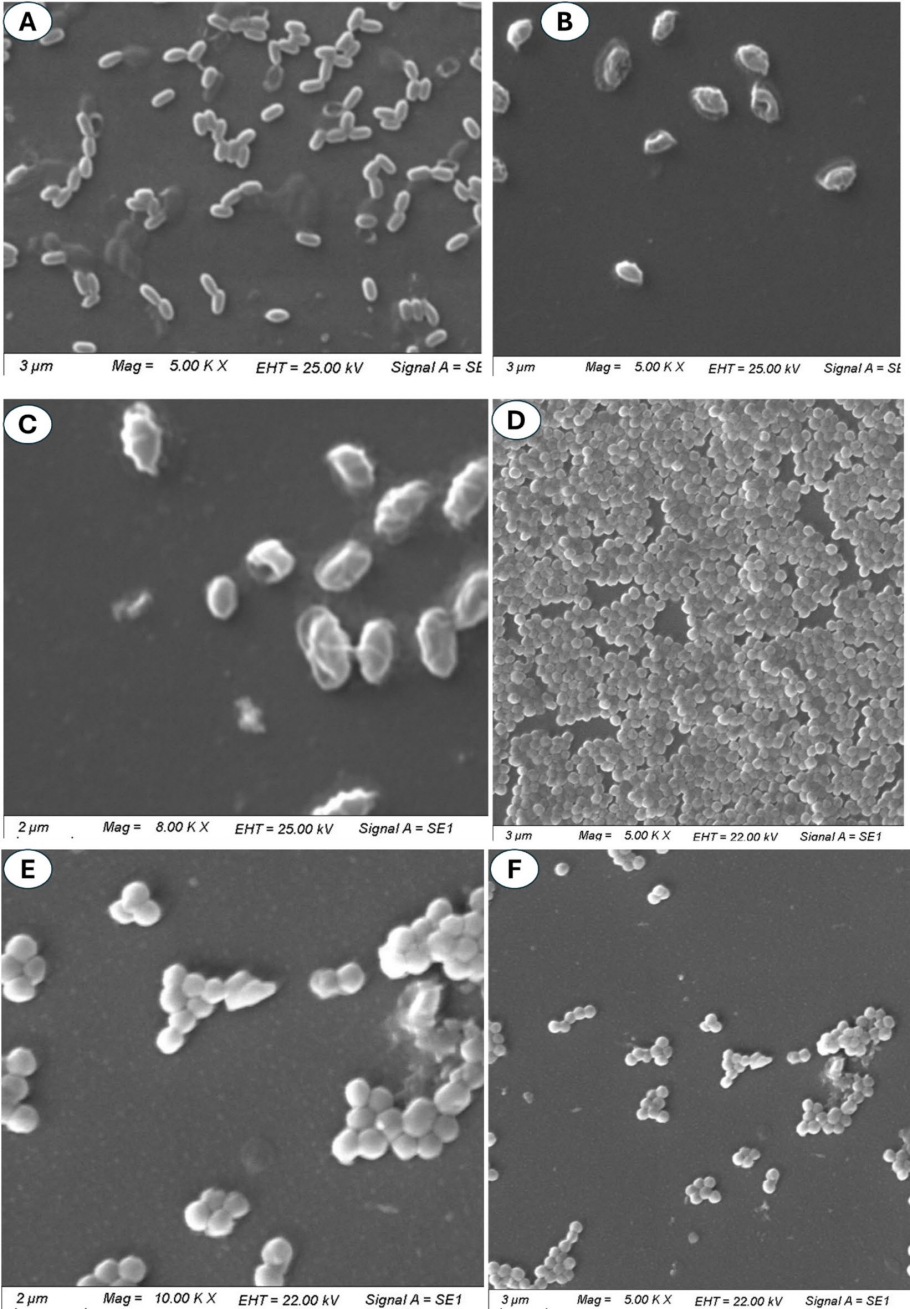
SEM image for *E. coli* and *Enterococcus faecalis* as the most affected bacterial cells before and after TiO_2_-NPs treatment showing morphological and structural changes.

To investigate the effect of biogenic TiO_2_-NPs on the morphology and structure of microbial cells, two bacterial strains represented as *E. coli* and *E. faecalis* as highly affected strains were selected for SEM analysis before and after treatment. As shown, the normal cells (without treatment) of *E. coli* appear rod-shaped, smooth, and intact. Moreover, the cells were uniform in size, the membrane was well-defined, there was no damage on the surface of the cells, and the cells were well-distributed without any cluster or deformation (Fig. 9A). In contrast, the surface of treated cells appeared rough and irregular. Some pitting on the cell surface may be due to NPs penetration. Moreover, the normal rod shape was distorted, shrinking, shriveling, and irregular due to membrane damage. Also, some treated cells appear fragmented, lysed, and contacted due to the cell membrane integrity and dysfunction of cell viability (Fig. 9B and C).

Regarding *E. faecalis*, the untreated cells (control) typically appear cocci, spherical, and smooth, with no signs of deformations. The cells were well-arranged and showed a healthy population without any stresses (Fig. 9D). Whereas the treated cells had irregular and rough surfaces (due to damage to the cell membrane), some cells were clustered (due to cellular stress caused by TiO_2_-NPs), shrinking, fragmented, and deformation of some treated cells (due to membrane integrity and oxidative stress) (Fig. 9E and F).

#### Antibiofilm

**Fig. 10:**
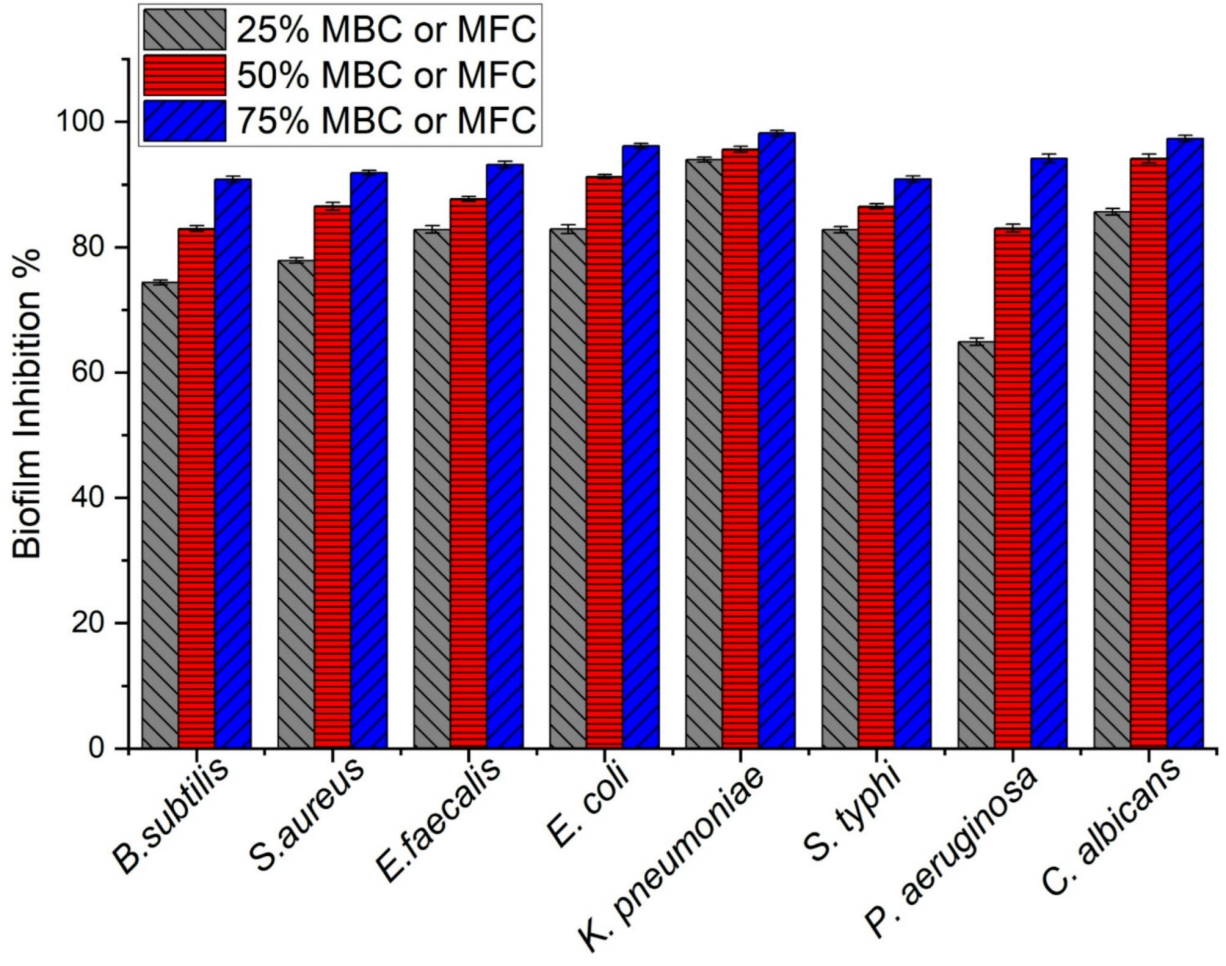
Antibiofilm activity of TiO_2_-NPs against pathogenic tested strains (G+, G−, and unicellular fungi) at concentrations of 25, 50, and 75% of MBC value for bacterial strains or MFC value for *C. albicans*.

The antibiofilm potential of TiO_2_-NP was then evaluated against the previously tested bacterial and unicellular strains at concentrations of 25%, 50%, and 75% of the MBC value detected above. The antibiofilm activity against the tested bacteria increased as the TiO_2_-NP concentration increased. With higher concentrations, such a dose-dependent effect of TiO_2_-NP on biofilm inhibition led to greater antibiofilm activity against the tested G + bacterial strains, particularly *E. faecalis*, which demonstrated the highest susceptibility among the three G + bacteria. At 25% of MBC, the anti-biofilm activity ranged from 74.37% for *B. subtilis* to 82.84% for *E. faecalis*. When the concentration was increased to 50% of MBC, the anti-biofilm activity improved, ranging from 82.97% against *B. subtilis* to 87.72% against *E. faecalis.* Notably, TiO_2_-NP exhibited its highest anti-biofilm activity at 75% of MBC, with *E. faecalis* being the most susceptible (93.20% inhibition), followed by *Staphylococcus aureus* (91.85% inhibition) and *B. subtilis* (90.82% inhibition) (Fig. 10).

For G- bacteria, TiO_2_-NP exhibited varying anti-biofilm activity against the tested G-ve bacteria, with the highest inhibition observed at 75% of MBC for all strains. Notably, *K. pneumoniae* was the most susceptible, with 98.20% inhibition at 75% of MBC, followed by *P. aeruginosa* (94.18%), *E. coli* (96.14%), and *S. typhi* (90.88%) (Fig. 10). These findings highlight the potential of TiO_2-_NP as an effective anti-biofilm agent against various G- bacterial strains, particularly at higher concentrations.

Against *Pseudomonas aeruginosa*, TiO_2_-NP demonstrated potent anti-biofilm activity, with inhibition percentages of 64.90%, 83.03%, and 94.18% at 25%, 50%, and 75% of the MBC, respectively. Similarly, for *Escherichia coli*, TiO_2_-NP exhibited strong anti-biofilm activity, with inhibition percentages of 82.89%, 91.25%, and 96.14% at 25%, 50%, and 75% of MBC, respectively, indicating a substantial reduction in biofilm formation at higher concentrations. For *Klebsiella pneumoniae*, TiO_2_-NP demonstrated remarkable anti-biofilm activity, with inhibition percentages of 93.95%, 95.61%, and 98.20% at 25%, 50%, and 75% of MBC, respectively. This suggests that even at lower concentrations, TiO_2_-NP was highly effective in inhibiting biofilm formation by *K. pneumoniae*. *Salmonella typhi* also exhibited susceptibility to the anti-biofilm activity of TiO_2_-NP, with inhibition percentages of 82.79%, 86.52%, and 90.88% at 25%, 50%, and 75% of MBC, respectively.

*C. albicans* is the most common causative agent of fungal infections spanning severe life-endangering conditions to relatively benign mucosal infections. Mucocutaneous candidiasis is classified as a non-genital and genitourinary disease. Oropharyngeal manifestations are often prevalent in non-genitourinary mucocutaneous candidiasis^90^. Herein, TiO_2_-NP exhibited potent anti-biofilm activity against such opportunistic fungus, with the inhibition percentage increasing as the concentration increased. At 25% of MBC, TiO_2_-NP demonstrated 85.64% inhibition of biofilm formation. When the concentration was increased to 50% of MBC, the anti-biofilm activity improved to 94.16%. Remarkably, TiO_2_-NP exhibited its highest anti-biofilm activity against *C. albicans* at 75% of MBC, with 97.34% inhibition of biofilm formation (Fig. 10).

A study showed that TiO_2_ nanoparticles at 500 µg/ml inhibited biofilm formation in both high and low-virulence *S. aureus* (MRSA) strains, indicating their potential as antibacterial agents^91^. Another study utilized TiO_2_-NPs combined with the antibiotic ceftazidime and cefotaxime against multidrug-resistant *Pseudomonas aeruginosa* isolated from respiratory mucus and Respiratory tract fluid samples. TiO_2_-NPs displayed antibiofilm effects at concentrations above 350 µg/ml. While the minimum inhibitory concentrations of TiO_2_-NPs were sixfold higher than the antibiotics alone, the combined application of antibiotics and TiO_2_-NPs synergistically enhanced the antimicrobial activity^92^.

## Materials and methods

### Actinobacterium used

The actinobacterium strain AMG31 was previously isolated from marine sediment collected from Marsa Allam, Red Sea, Egypt, and utilized to produce exopolysaccharide. The identification of this strain was achieved based on morphological, physiological, biochemical identification, and molecular based on sequencing of 16 S rRNA gene as *Streptomyces vinaceusdrappus* AMG31 and added to the GenBank database with accession number OR793047^93^.

### *S. vinaceusdrappus* AMG31-mediated biogenic synthesis of TiO_2_-NPs

**Fig. 11:**
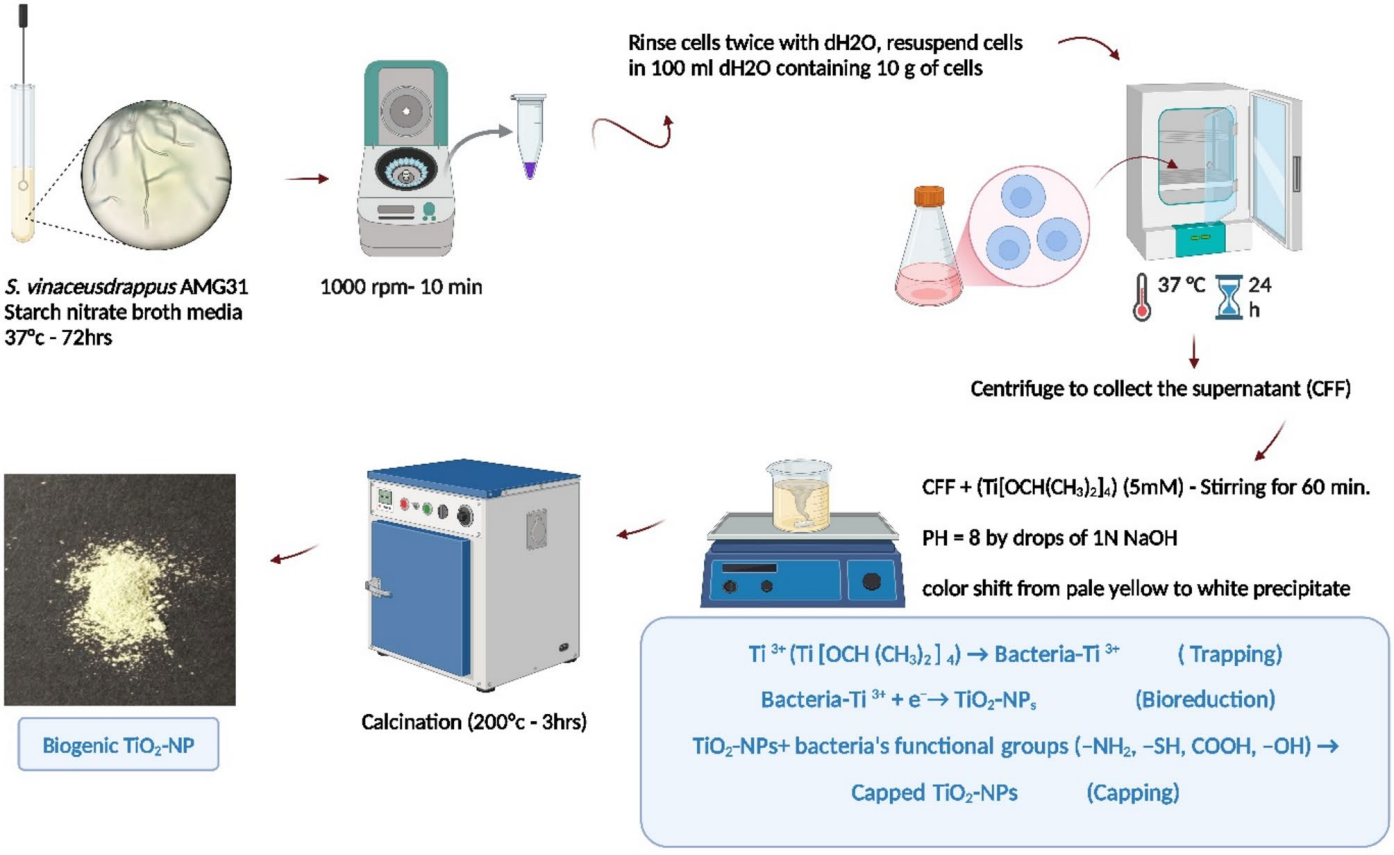
Flowchart showing different steps for synthesis of TiO_2_-NPs using *S. vinaceusdrappus* AMG31.

A single colony of *S. vinaceusdrappus* AMG31 was inoculated into starch nitrate broth media and incubated at 37 °C for 72 h. Subsequently, the inoculated media underwent centrifugation at 1000 rpm for 10 min to collect the cells. These cells were then rinsed twice with sterilized distilled water (dH_2_O) before resuspending in approximately 100 ml of distilled water containing 10 g of cells. The previous mixture was then incubated again at 37 °C for 24 h. After the incubation period ended, the mixture was centrifuged to collect the supernatant (cell-free filtrate), which was utilized as a biocatalyst for the biosynthesis of TiO_2_-NPs. For this purpose, the filtrate was mixed with titanium isopropoxide (Ti[OCH(CH_3_)_2_]_4_) under stirring conditions for 1 h to achieve a final concentration of 5 mM^48^. The pH of the mixture was adjusted to 8 by dropwise addition of 1 N NaOH. A shift in color from pale yellow to white precipitate signified the successful synthesis of TiO_2_-NPs. After 24 h of incubation, the white precipitate was harvested, rinsed three times with high-purity Milli-Q water, and dried in an oven at 200 °C for 3 h (Fig. 11).

### Characterization of the biosynthesized TiO_2_-NP

The formation of TiO_2_-NPs was first evident from the color change to white in the cell-free filtrate after adding the metal precursor. The functional groups present in the bacterial cell-free filtrate and the synthesized TiO_2_-NPs were examined using Fourier transform infrared (FT-IR) spectroscopy (Cary-660 model). For this analysis, 10 mg of TiO_2_-NPs or 3 ml of the cell-free filtrate was mixed thoroughly with KBr and pressed into a disk. Subsequently, the disk was scanned over the wavenumber range of 400–4000 cm^− 1^^94^.

The crystalline or amorphous nature of the synthesized TiO_2_-NPs was examined using X-ray diffraction (XRD) analysis on a PANalytical-X’Pert-Pro-MRD instrument. The X-ray source was CuKα radiation with a wavelength of 1.54 Å, operated at 30 mA current and 40 kV voltage. The XRD scan was performed at 2θ ranges of 10–80°. The average crystallite size of the biosynthesized TiO_2_-NPs was calculated from the XRD data using the Debye-Scherrer equation^95^ as follows:

1 $$\:Average\:size=\:\frac{0.94\:x\:1.54}{\beta\:\:cos\theta\:}$$

Where 0.94 is the constant of Scherrer, 1.54 is the wavelength of the X-ray, β is the half intensity, and θ is the angle of Bragg’s diffraction.

For transmission electron microscopy (TEM, JEOL, Ltd-1010, Tokyo, Japan) analysis, the as-formed powder of TiO_2_-NPs was first suspended in high-purity Milli-Q water under sonication. The drops of this suspension were then dried on a TEM grid before examination. The dried nanoparticle powder was separately analyzed using a scanning electron microscope (JEOL, JSM-6360LA, Tokyo, Japan) to study the surface morphology. Concurrently, energy-dispersive X-ray spectroscopy (EDX) and SEM were employed to determine the chemical composition of the nanoparticles.

For scanning electron microscopy (SEM) analysis, the biosynthesized TiO_2_-NPs were loaded onto an SEM holder, coated with gold under vacuum, and imaged at 20 kV using a JEOL JSM-6360LA instrument (Tokyo, Japan). Concurrently, the elementary qualitative and quantitative mapping of the nanoparticles was performed by energy-dispersive X-ray spectroscopy (EDX) coupled with the SEM apparatus^50^. Dynamic light scattering (DLS) was used to investigate the nanoparticle’s hydrodynamic sizes and size distribution in colloidal solutions. For this, the biosynthesized TiO_2_-NPs were suspended in high-purity water to avoid the formation of shadows and anomalous peaks during particle scattering. Additionally, the surface charge of the synthesized nanoparticles was analyzed by measuring their zeta potential using a Malvern Zeta-sizer (Nano-ZS, Malvern, UK)^96^. The optical properties of biogenic TiO_2_-NPs were investigated by UV-Vis spectroscopy (JENWAY-6306, Staffordshire, UK) for detecting the maximum surface plasmon resonance (SPR). Approximately 2 mL of synthesized solution was transferred to a spectrophotometric cuvette to measure their absorbance in 200–800 nm ranges.

### Biomedical applications

#### Antioxidant activity

##### DPPH

The free radical scavenging activity of TiO_2_-NP was measured using DPPH (1,1-diphenyl-2-picryl hydrazyl). In brief, a 0.1 mM solution of DPPH in ethanol was prepared. This solution (1 ml) was added to 3 ml of TiO_2_-NP in ethanol at different concentrations (1.95–1000 µg/ml). The mixtures were shaken and allowed to stand for 30 min before measuring absorbance at 517 nm using a spectrophotometer (UV-VIS milton roy). Ascorbic acid was used as a reference standard. The percentages of DPPH scavenging were calculated using the following formula^97^.

2 $$DPPH{\text{ }}Scavenging{\text{ \% = }}\frac{{{A_0} - {{\text{A}}_{\text{1}}}}}{{{A_0}}} \times {\text{100}}$$

A_0_ and A_1_ absorbance of ascorbic acid and TiO2-NP treatment, respectively.

##### ABTS

The ABTS^•+^ radical scavenging activity of the TiO_2_-NP was determined using the method described by González et al.^98^. The ABTS radical cation (ABTS^•+^) was generated by reacting a 7 mM 2,20-azino-bis(3-ethylbenzothiazoline-6-sulphonicacid) (ABTS) stock solution with 2.45 mM K_2_S_2_O_8_ and the mixture was allowed to stand in the dark at room temperature. The assay mixture comprised 0.07 ml of TiO_2_-NP and 3 ml of the diluted ABTS^•+^ solution. After a 6-minute incubation period, the absorbance was measured at 734 nm. The radical scavenging capacity was expressed relative to gallic acid’s standard antioxidant reference.

3 $$ABT{S^{ \cdot +}}{\text{ }}scavenging{\text{ }}\% =\frac{{A - B}}{A} \times 100$$

Where A is the absorbance of the solution at zero time (negative control), and B is the absorbance after 6 min of adding TiO_2_-NPs.

##### TAC

The phosphomolybdenum method was employed to evaluate the total antioxidant capacity (TAC) of TiO_2_-NP. A solution containing 0.5 mg/ml of TiO_2_-NP was mixed with 0.6 M H_2_SO_4_, 28 mM NaH_2_PO_4_, and 4 mM ammonium molybdate. A control solution with only the reagent was prepared for comparative analysis. These mixtures were then heated at 95 °C for 150 min^99^. Upon cooling down to ambient temperature, their absorbance was recorded at 630 nm, and the findings were presented as ascorbic acid equivalent (AAE) in µg/mg.

##### FRAP

The effect of solvent polarity on the ferric-reducing antioxidant power of TiO_2_-NP was evaluated using the K_3_Fe(CN)_6_ and C_2_HCl_3_O_2_ method, as described by Benzie & Strain^100^, with modifications and adaptations for a microplate assay^101^. Eppendorf tubes were labeled, and 40 µl of TiO_2_-NP was introduced to each tube, then adding 50 µl of 0.2 mol/l (Na_2_HPO_4_.2H_2_O) buffer, 50 µl of 1% K_3_Fe(CN)_6_, and 50 µl of 10% C_2_HCl_3_O_2_. The mixture was spun at 3,000 rpm for 10 min. After centrifugation, 165 µl of the supernatant from each TiO_2_-NP was transferred to a 96-well plate, followed by the addition of 33.3 µl of 1% FeCl_3_. The absorbance was measured at 630 nm, and the results were presented as ascorbic acid equivalent (AAE) in µg/mg.

#### Wound healing

The HFB4 cell line of human normal skin fibroblasts received from VACSERA Company, Cairo, Egypt, was used to examine the wound healing activity of synthesized TiO_2_-NPs. The HFB4 cells were seeded into multi-well plates and allowed to grow until forming a confluent monolayer, ensuring all cultures were fully confluent at the beginning, which was crucial. A straight scratch was made to simulate a wound using a yellow pipette tip angled at approximately 30 degrees. Keeping the pipette tip at an angle helped maintain a narrow scratch width, enabling imaging of both wound edges under a 10x objective lens^102^. The following equation calculated the rate of migration (RM):

4 $${\text{RM}}=\frac{{Wi - Wf}}{T}$$

Where Wi is the width average of the initial wound (µm), Wf is the final wound’s (µm) width average, and T is time. On the other hand, the wound closure (WC) percentages were calculated as follows:

5 $${\text{WC\% }}=\frac{{A{t_0} - A{t_{\Delta t}}}}{{A{t_0}}} \times 100$$

Where At_0_ is the wound area at an initial time, and At_Δt_ is the wound area after n hours.

Moreover, the difference between initial and final area (AD) was calculated as follows:

6 $${\text{AD \% }}={{\text{A}}_{\text{i}}} - {{\text{A}}_{\text{f}}}$$

Where A_i_ is the initial area, and A_f_ is the final area.

#### Hemocompatibility assay

##### Anticoagulation test

To evaluate anticoagulant activity, classic coagulation assays, including activated partial thromboplastin time (APTT) and prothrombin time (PT), were performed to study TiO_2_-NPs using a systematic method of assessment of blood clotting properties. TiO_2_-NPs applied had concentrations of 0, 25, 50 and 75 µg/ml, heparin was used as the positive control. Citrated plasma (3:1) was mixed with TiO_2_-NP solution, and the mixture was kept in the incubator at 37 °C for 3 min. For PT testing, 0.20 ml of reagent was pre-incubated for 3 min at 37 °C and added. While for APTT testing, 0.10 ml of reagent and 0.10 ml CaCl_2_ (0.025 mol/l) were sequentially added and incubated for 5 min at 37 °C, with clotting time noted to examine the blood anticoagulant properties^103^.

#### RBCs cell membrane integrity

Human red blood cells (RBCs) were subjected to three washings in 150 mM NaCl and centrifuged at 2500 RPM for 10 min and then resuspended in Phosphate Buffer Saline solution (pH 7.4) to yield a 2% RBC solution. The TiO_2_-NPs were assessed at concentrations of 25, 50, 100, 200, 400, 600, 800, and 1000 µg/ml, employing full hemolysis and the isotonic solution respectively as positive and negative control. RBC’s solution and a serial dilution of TiO_2_-NPs were combined to yield a final volume of 1 ml. The solution was then placed at 37 °C for an hour, spun at 2500 for 15 min, and the optical density of the supernatant fluid was measured at 546 nm^104^, with experiments performed in triplicate and mean ± SD determined.

7 $${\text{Hemolytic \% = }}\frac{{{\text{A}}{{\text{b}}_{{\text{treatment}}}} - {\text{A}}{{\text{b}}_{{\text{blank}}}}}}{{{\text{A}}{{\text{b}}_{{\text{positive control}}}}}} \times {\text{100}}$$

Ab is the absorbance of the supernatant fluid measured at 546 nm, blank is the isotonic solution, and positive control is full hemolysis.

#### Cytotoxicity

To assess the cytotoxic impact of TiO_2_-NP, three different cell lines were utilized: the WI38 normal fibroblast cell line, the Caco-2 colorectal adenocarcinoma cell line, and the PANC-1 pancreatic cancer cell line. These cell lines were procured from VACSERA Company, Cairo, Egypt. The assay was carried out using the MTT method. Each cell type was grown in separate 96-well plates, with approximately 10,000 cells per well immersed in a growth medium solution. After an initial 24-hour period, the cells were exposed to varying concentrations of TiO_2_-NP (31.25–1000 µg/ml) while control wells received a DMSO solution. The plates were then incubated for an additional 24 h in a controlled environment maintained at 37 °C and 5% CO_2_ levels. Subsequent to incubation, the medium was carefully removed from each well, and the cells were rinsed with distilled water to eliminate any residual nanoparticles. A 1% crystal violet staining solution was applied to the cells for 30 min, allowing them to absorb the dye. After discarding the stain, a 30% glacial acetic acid solution was introduced to the wells, and the plates were gently agitated. Each well’s absorbance was then read at 490 nm using a microplate ELISA reader^105^. To ensure reliable results, these experiments were conducted in triplicate.

8 $${\text{Cell viability \% }}=\frac{{{\text{Absorbance of treated cells}}}}{{{\text{Absorbance of control}}}} \times {\text{100}}$$

#### Antidiabetic

##### α-Amylase Inhibition

The inhibitory capability of TiO_2_-NP against the α-amylase enzyme was assessed utilizing the 3,5-dinitrosalicylic acid (DNS) method. Tested concentrations of TiO_2_-NPs, ranging from 1.95 to 1000 µg/ml, were evaluated and compared to acarbose, a known α-amylase inhibitor, which served as a positive control at the same concentration range. The absorbance readings were obtained at a wavelength of 540 nm using a UV-visible Biosystem 310 spectrophotometer^106^. The α-amylase inhibition percentages were calculated using the following equation:

9 $${\text{Enzyme inhibition \% = }}\frac{{{\text{A}}{{\text{b}}_{{\text{control}}}} - {\text{A}}{{\text{b}}_{{\text{treatment}}}}}}{{{\text{A}}{{\text{b}}_{{\text{control}}}}}} \times {\text{100}}$$

Where Ab is the absorbance.

To determine the IC_50_ values, which represent the concentration of TiO_2_-NP required to inhibit 50% of the α-amylase activity, a graph was plotted illustrating the percentage of α-amylase inhibition against the corresponding tested TiO_2_-NP concentrations.

##### α-Glucosidase Inhibition

To evaluate the ability of TiO_2_-NP (1.95–1000 µg/ml) to inhibit the α-glucosidase enzyme. The inhibitory activity of TiO_2_-NP was compared to acarbose, which served as a positive control^107^. Absorbance readings are measured at 405 nm wavelength using a Biosystm 310 Plus spectrophotometer, and the inhibition percentages were detected using Eq. 8. To determine the IC_50_ value, a regression equation is derived by plotting the percentage of inhibition against the corresponding TiO_2_-NP concentrations tested, spanning from 1.95 to 1000 µg/ml.

#### Antimicrobial activity

The antimicrobial activity of TiO_2_-NP was evaluated using the agar well diffusion method against a broad spectrum of bacterial and fungal strains. The test was achieved against ATCC (American Type Culture Collection) for bacterial strains and unicellular fungi, whereas multicellular fungi were purchased from Assiut University Mycological Centre (AUMC). The antibacterial activity was achieved against Gram-positive strains (*Bacillus subtilis* ATCC 6633, *Staphylococcus aureus* ATCC 6538, and *Enterococcus faecalis* ATCC 29212) and Gram-negative strains (*Escherichia coli* ATCC 8739, *Klebsiella pneumoniae* ATCC 13883, *Pseudomonas aeruginosa* ATCC 90274, and *Salmonella typhi* ATCC 6539) on Mueller-Hinton agar media. Additionally, the antifungal activity was assessed against *Aspergillus niger* (AUMC 14260), *Mucor circinelloides* (AUMMC 11656), *Trichoderma harzianum* (AUMC 5408), *Penicillium glabrum* (OP694171) (AUMC15597), and *Candida albicans* (ATCC 10221) on Sabouraud dextrose agar. Gentamicin (positive control) was employed as the control drug for bacterial inhibition screening, while Fluconazole (positive control) served as the antifungal control.

The inoculum suspension was appropriately adjusted for the standard broth dilution method, and the agar plates were inoculated within 15 min. The dried agar surface was streaked in three directions. After allowing the agar to dry for 15 min, a sterile cork borer with a diameter of 7 mm was used aseptically to create wells in the agar. TiO_2_-NP was suspended in DMSO at 1 mg/ml, and 100 µl of the stock solution was dispensed into each well compared to the same positive control and negative control (DMSO) concentration. The plates were then incubated for 24 h for bacteria and unicellular fungi and 72 h for multicellular fungi. The zones of inhibition were measured to the nearest whole millimeter at the point where significant growth reduction was observed^108^.

For MIC (minimum inhibitory concentrations), MBC (minimum bactericidal concentrations), and MFC (minimum fungicidal concentrations), the detection was performed using the microplate assay method by investigating the activity of different TiO_2_-NPs concentrations (400, 200,100,50, 25, and 12.5 µg/ml). The assay was achieved following the guidelines established by the Clinical and Laboratory Standards Institute (CLSI)^109^. The morphological changes of highly affected bacterial cells, *E. coli* (Gram-negative) and *Enterococcus faecalis* (Gram-positive) were investigated under scanning electron microscopy.

#### Antibiofilm

The impact of TiO_2_-NP on biofilm formation was assessed using 96-well polystyrene flat-bottom plates against all tested bacterial strains and *C. albicans*. Briefly, 300 µl of trypticase soy yeast broth (TSY) containing 10^6^ CFU/ml was inoculated with sub-lethal concentrations of 75%, 50%, and 25% of the MBC/MFC of TiO_2_-NP. After incubating for 48 h at 37 °C, the biofilm formed on the plates was stained for 15 min with a 0.1% aqueous crystal violet solution. The excess stain was removed by rinsing with sterile distilled water. Subsequently, 250 µl of 95% ethanol was added to each well to solubilize the dye taken up by the cells. The absorbance at 570 nm was measured using a microplate reader after 15 min, which quantified the biofilm formation^110^.

10 $${\text{Biofilm inhibition \% =}}\frac{{{{\text{A}}_{\text{S}}} - {{\text{A}}_{\text{B}}}}}{{{{\text{A}}_{\text{C}}} - {{\text{A}}_{\text{B}}}}} \times {\text{100}}$$

Where A is the absorbance at 570 nm, A_S_ is the absorbance of the sample (media + test organism + TiO_2_-NPs at 25%, 50%, or 75% MBC value), A_B_ is the absorbance of blank (media only), and A_C_ (media + test organism).

### Statistical analysis

SPSS (version 18, USA) was used for data analysis. The Shapiro-Wilk, Equal Variance, and One-Way Analysis of Variance (ANOVA) tests were performed. Following the ANOVA test was conducted.

## Conclusion

The biosynthesis of TiO₂-NPs utilizing the marine actinobacterium *Streptomyces vinaceusdrappus* AMG31 brought noteworthy experimental findings. Characterization showed well-dispersed, spherical NPs with sizes ranging from 10 to 50 nm (average 23.47 ± 1.107 nm) in the highly crystalline anatase phase. The actinobacterial TiO_2_-NPs showed considerable multifunctional properties, exhibiting marked antioxidant activity with maximum DPPH and ABTS scavenging percentages of 87.9% and 88.2%, respectively, at 500 µg/ml, while also confirming moderate wound healing activities, having achieved 66.6% closure as opposed to 62.6% in controls after 48 h. The investigations on blood compatibility proved optimal hemocompatibility with low hemolytic activity and a reasonable dose of anticoagulant effect. Notably, TiO₂-NPs exhibited selective cytotoxic activity against several cancer cell lines, inhibiting proliferation at 74.1 µg/ml for Caco-2 and 71.0 µg/ml for PANC-1 while showing relatively lesser effects on normal WI38 cells with an IC₅₀ of 153.1 µg/ml. For antimicrobial activity, TiO₂-NPs showed superior efficacy against G + ve bacteria (inhibition zones: 29–37 mm vs. 22–29 mm for gentamicin), with *E. faecalis* being particularly susceptible (MIC: 12.5 µg/ml, MBC: 25 µg/ml, MBC/MIC: 2). Against G-ve bacteria, *E. coli* showed notable sensitivity (MIC: 6.25 µg/ml, MBC: 12.5 µg/ml, MBC/MIC: 2). The nanoparticles demonstrated substantial antifungal activity against various species, particularly *A. niger* (MIC: 6.25 µg/ml, MFC: 50 µg/ml) and *C. albicans* (MIC: 6.25 µg/ml, MFC: 25 µg/ml). Furthermore, TiO₂-NPs exhibited robust antibiofilm activity, achieving 90.8–93.2% inhibition for G + ve bacteria, 90.9–98.2% for G-ve bacteria, and 97.3% inhibition for *C. albicans* at 75% MBC/MFC concentrations. The limitations of this investigation also include the requirement for thorough in-vivo toxicity experiments and testing in vivo efficacy for clinical relevance. Future studies should aim at the mechanistic basis for resistance developing in microorganisms, rationalizing synthesis for industrial up-scaling, and targeting synergistic approaches for clinical translatability.

## Electronic supplementary material

Below is the link to the electronic supplementary material.

Supplementary Material 1

## Data Availability

The datasets used and/or analysed during the current study available from the corresponding author on reasonable request.
