# Supplementary material for: Multifaceted biomedical applications of biogenic titanium dioxide nanoparticles fabricated by marine actinobacterium Streptomyces vinaceusdrappus AMG31
Source: Sci Rep. 2025 Jun 23;15:20244. doi: 10.1038/s41598-025-00541-1 (PMC12185696; doi:10.1038/s41598-025-00541-1)
Supplement: Supplementary file 1 — Supplementary Material 1 [file 41598_2025_541_MOESM1_ESM.docx]

**Multifaceted Biomedical Applications of Biogenic Titanium Dioxide Nanoparticles Fabricated by Marine Actinobacterium *Streptomyces Vinaceusdrappus* AMG31**

**Ahmed Ghareeb** ^1^**, Amr Fouda** ^2*^**, Rania M. Kishk** ^3^ **and Waleed M. El Kazzaz** ^1^

^1^ Botany and Microbiology Department, Faculty of Science, Suez Canal University, Ismailia 41522, Egypt.

^2^ Botany and Microbiology Department, Faculty of Science, Al-Azhar University, Nasr City, Cairo 11884, Egypt.

^3^ Microbiology and Immunology Department, Faculty of Medicine, Suez Canal University, Ismailia 41522, Egypt.

^*^ Corresponding author: Amr Fouda, [amr_fh83@azhar.edu.eg](mailto:amr_fh83@azhar.edu.eg) (ORCID Number: [https://orcid.org/0000-0003-3840-7837](https://www.scopus.com/redirect.uri?url=https://orcid.org/0000-0003-3840-7837&authorId=57194940078&origin=AuthorProfile&orcId=0000-0003-3840-7837&category=orcidLink))

Table S1. Calculation of crystallite size for each XRD peak using the Debye-Scherrer equation.

| **2θ (°)** | **FWHM (°)** | **Crystallite Size (nm)** |
| --- | --- | --- |
| 25.57 | 0.227º | 35.89 |
| 38.21 | 0.202º | 37.31 |
| 48.25 | 0.304º | 38.52 |
| 54.42 | 0.265º | 33.71 |
| 55.45 | 0.314º | 28.58 |
| 63.16 | 0.379º | 24.60 |
| 68.98 | 0.307º | 27.08 |
| 70.74 | 0.198º | 36.92 |
| 75.53 | 0.209º | 37.21 |
| Average crystallite size (nm) | | 33.3 |


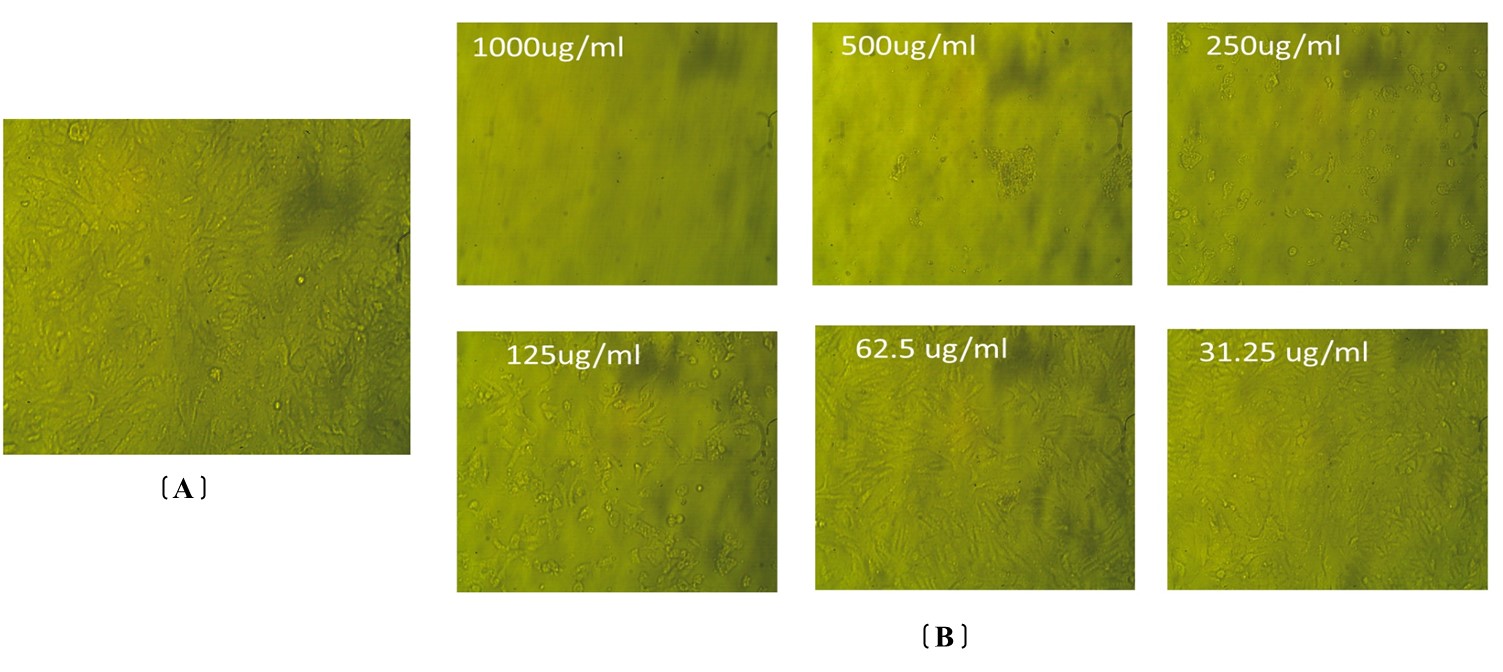


**Figure S1**: Morphological changes and size variation of WI-38 cells before and after TiO_2_-NP treatment (A) Untreated WI38 cells showing normal morphology and size (B) WI-38 cells treated with 31.25-1000 μg/mL TiO_2_-NP


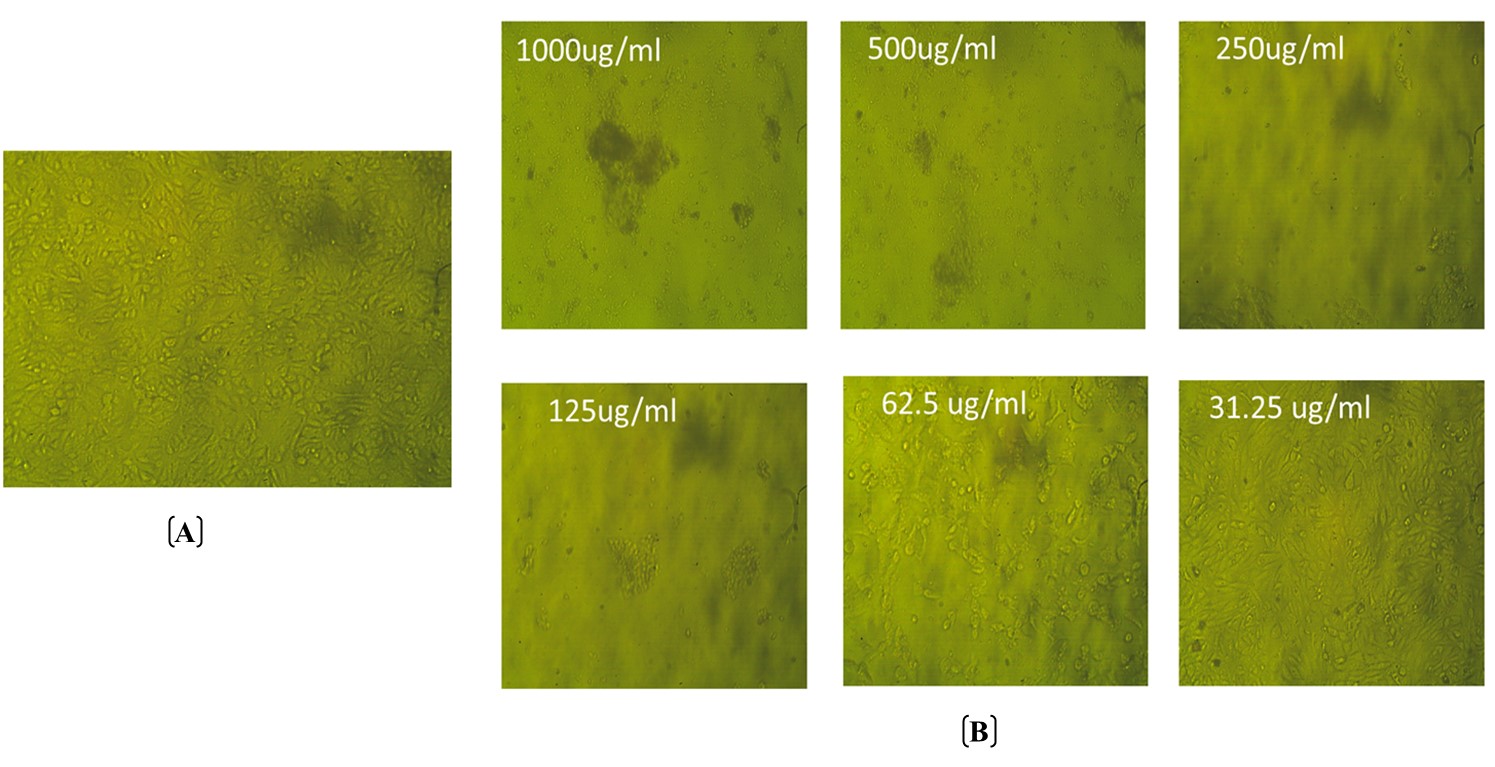


**Figure S2**: Morphological changes and size variation of Caco-2 cancer cell line before and after TiO_2_-NP treatment (A) Untreated Caco-2 cancer cell line showing normal morphology and size (B) Caco-2 cancer cell line treated with 31.25-1000 μg/mL TiO_2_-NP showing changes in morphology and size


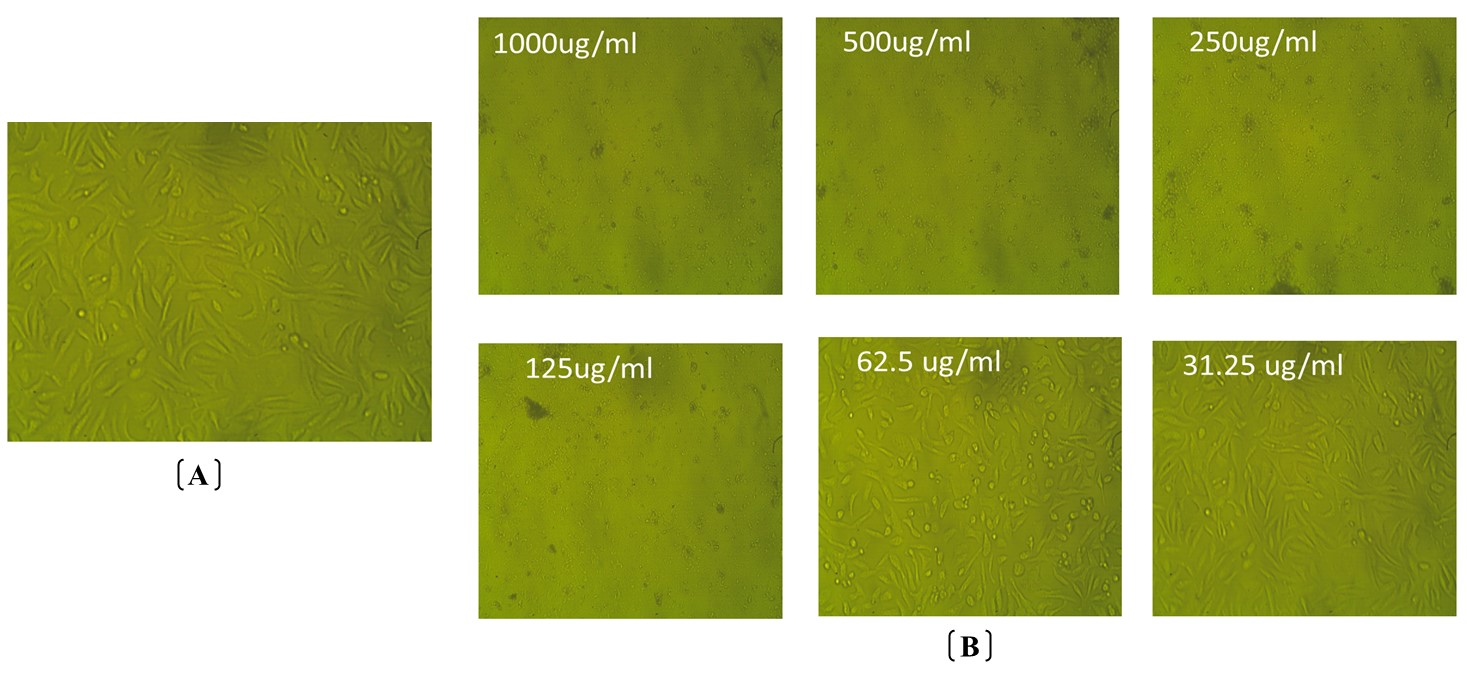

**Figure S3**: Morphological changes and size variation of PANC-1 cancer cell line before and after TiO_2_-NP treatment (A) Untreated PANC-1 cancer cell line showing normal morphology and size (B) PANC-1 cancer cell line treated with 31.25-1000 μg/mL TiO_2_-NP showing changes in morphology and size


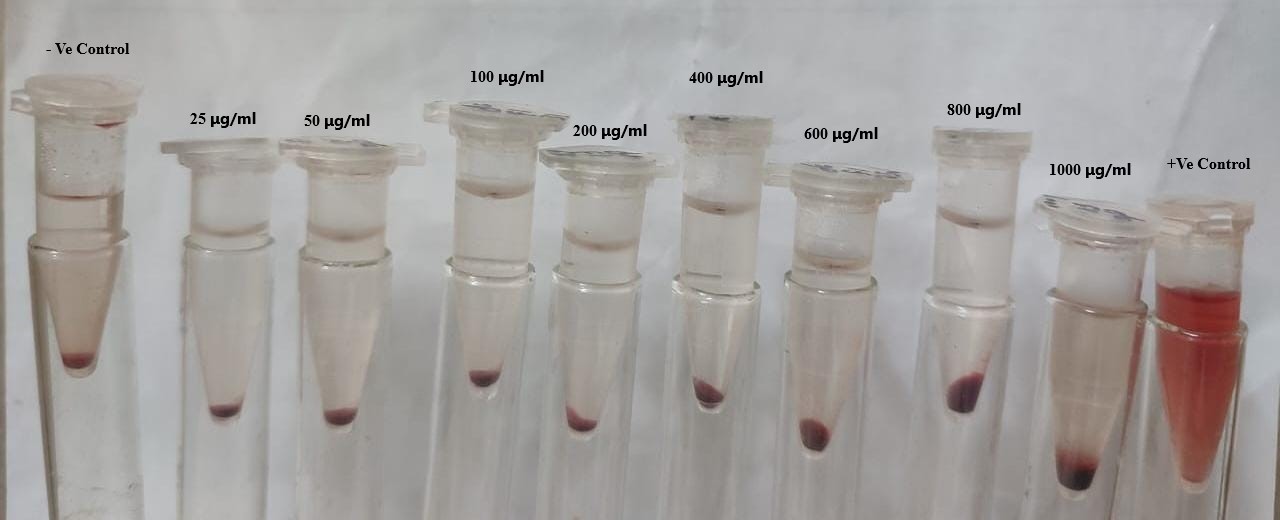


**Figure S4**: Concentration gradient of TiO_2_-NPs and its effects on RBCs hemolysis
